# NanoAmpli-Seq: a workflow for amplicon sequencing for mixed microbial communities on the nanopore sequencing platform

**DOI:** 10.1093/gigascience/giy140

**Published:** 2018-11-23

**Authors:** Szymon T Calus, Umer Z Ijaz, Ameet J Pinto

**Affiliations:** 1 School of Engineering, University of Glasgow, Glasgow, G12 8QQ, United Kingdom; 2 Department of Civil & Environmental Engineering, Northeastern University, 417 SN, 360 Huntington Avenue, Boston, MA, USA

**Keywords:** amplicon sequencing, nanopore sequencing, *de novo* analyses, sequencing accuracy

## Abstract

**Background:**

Amplicon sequencing on Illumina sequencing platforms leverages their deep sequencing and multiplexing capacity but is limited in genetic resolution due to short read lengths. While Oxford Nanopore or Pacific Biosciences sequencing platforms overcome this limitation, their application has been limited due to higher error rates or lower data output.

**Results:**

In this study, we introduce an amplicon sequencing workflow, i.e., NanoAmpli-Seq, that builds on the intramolecular-ligated nanopore consensus sequencing (INC-Seq) approach and demonstrate its application for full-length 16S rRNA gene sequencing. NanoAmpli-Seq includes vital improvements to the INC-Seq protocol that reduces sample processing time while significantly improving sequence accuracy. The developed protocol includes chopSeq software for fragmentation and read orientation correction of INC-Seq consensus reads while nanoClust algorithm was designed for read partitioning-based *de novo* clustering and within cluster consensus calling to obtain accurate full-length 16S rRNA gene sequences.

**Conclusions:**

NanoAmpli-Seq accurately estimates the diversity of tested mock communities with average consensus sequence accuracy of 99.5% for 2D and 1D^2^ sequencing on the nanopore sequencing platform. Nearly all residual errors in NanoAmpli-Seq sequences originate from deletions in homopolymer regions, indicating that homopolymer aware base calling or error correction may allow for sequencing accuracy comparable to short-read sequencing platforms.

## Background

Amplicon sequencing, particularly sequencing of the small subunit rRNA (SSU rRNA) gene and internal transcribed spacer regions, is widely used for profiling of microbial community structure and membership [1–4]. The wide-scale application of amplicon sequencing has been driven mainly by the ability to multiplex 100s of samples on a single sequencing run and obtain millions of sequences of target communities on high-throughput sequencing platforms [1, 4]. The primary limitation of these commonly used technologies (e.g., Illumina's MiSeq, Ion Torrent PGM) is that their read lengths are short, ranging from 150 to 400 bp [5]. While excellent at bulk profiling of microbial communities through multiplexed deep sequencing, short read lengths are limited in the taxonomic resolution of sequenced reads and, more so, are not amenable to robust phylogenetic analyses to assess the relationship between sequences originating from unknown microbes with those in publicly available databases. An important effect of the proliferation in short read sequencing applications has been a decrease in the rate at which long higher-quality sequences, particularly of SSU rRNA genes, are being deposited in public databases. This effect is to some extent being mitigated through assembly and curation of near full-length SSU rRNA genes from metagenomic datasets [6–9] and will continue to be mitigated with novel approaches for SSU rRNA sequencing using synthetic long read approaches [10].

The introduction of single-molecule sequencing platforms, such as Pacific Bioscience's (PacBio's) single-molecule real-time sequencing (SMRT) and single-molecule sensing technologies on the Oxford Nanopore Technologies (ONT) MinION platform, has opened the possibility of obtaining ultra-long reads [5, 11]. While sequencing throughput and raw data quality of long-read single-molecule sequencing approaches are yet to rival that of short read platforms, the ability to obtain ultra-long reads can overcome several limitations of the latter [12]. For instance, long-read sequencing combined with various error correction approaches [13, 14] has been used to obtain high-quality single contig microbial genomes [14] or increase assembly quality of previously sequenced but fragmented eukaryotic genomes assemblies [15, 16], which was not feasible using short-read approaches. Long-read sequencing capabilities have also been recently leveraged to sequence near full-length SSU rRNA genes (e.g., 16S rRNA) [17–21] or even the entire *rrn* operon [20, 22].

A majority of the studies utilizing either the SMRT or nanopore sequencing platforms have restricted their data analyses efforts to sequence classification due to the fact that widely used sequence classifiers are tolerant of high sequencing error rates [23, 24]. However, these classification-only approaches are limited in their ability to differentiate between closely related sequences, risk false detections (i.e., read incorrectly classified at the family or genus levels due to high error rates), and are unable to identify organisms that are not represented in the reference databases. In contrast, some studies have gone beyond sequence classification by using consensus sequence construction to improve overall sequence accuracy. The consensus sequence creation efforts thus far can be categorized into two approaches. The first approach involves mapping raw, noisy reads to custom or publicly available reference databases (i.e., SILVA) [25]. Subsequently, reads mapping to the same reference sequence are then used for the semi-automated or manual construction of a consensus sequence using overlapping alignments [20, 22]. While this approach does result in improved accuracy of the consensus sequence, clustering of reads based on mapping of noisy reads to reference databases has significant limitations. First, incorrect mapping to a reference database is likely due to high error rates of raw nanopore reads. Second, the reliance on a reference database ensures that reads originating from organisms not represented in the reference database are typically discarded as these could lead to clustering errors during operational taxonomic unit (OTU) construction. The more robust alternative towards high accuracy consensus sequence generation would be a completely *de novo* approach, i.e., generation of a consensus sequence without the use of any reference database.

To our knowledge, there are three reports of *de novo* data processing to reduce error rate from long-read sequencing of amplicons from mixed microbial communities [17, 21, 26]. Both Singer et al. [26] and Schloss et al. [17] utilized the circular consensus sequencing approach of SMRT sequencing coupled with a range of quality filtering (i.e., mismatches to primer, quality scores) and sequence clustering (i.e., pre-cluster) to generate consensus sequences from reads clustered into OTUs and achieved error rates of 0.5% [26] and 0.027% [17] for full-length 16S rRNA gene sequencing libraries. For the latter effort [17], the number of OTUs in the processed data were also highly similar to the theoretical number of OTUs in tested mock communities, indicating that the application of this protocol for naturally derived mixed microbial communities is likely to result in robust diversity estimates. Li et al. [21] developed the intramolecular-ligated nanopore consensus sequencing (INC-Seq) protocol for consensus-based error correction of nanopore sequencing reads with a median accuracy of 97%–98%. The INC-Seq workflow involves amplicon concatemerization to link multiple identical copies of the same amplicon on a single DNA molecule, sequencing of the concatemerized molecules using 2D sequencing chemistry on the nanopore sequencing platform, followed by consensus-based error correction after aligning the physically linked concatemers on each sequenced DNA strand. By using this approach, Li et al [21] were able to increase the median sequence accuracy of processed reads to 97%–98%. While this significant improvement allowed for taxonomic classification of sequences to the species level, it did not allow for sequence clustering for diversity estimation due to residual median error rates of approximately 2%–3%.

In this study, we leverage and expand on the INC-Seq protocol developed by Li et al. [21] to provide a complete workflow for amplicon sequencing and *de novo* data processing called NanoAmpli-Seq. This protocol was applied to near full-length 16S rRNA gene of mock communities that resulted in high-quality sequences with a mean sequence accuracy of 99.5 ±0.08%. The current version of NanoAmpli-Seq includes modifications to the library preparation protocol for INC-Seq and fixes a key issue with INC-Seq consensus sequences while adding a novel read partitioning-based sequence clustering approach. These improvements result in an accurate estimation of diversity of mixed microbial communities and results in higher sequence accuracy by allowing within-OTU sequence alignment and consensus calling. Further, we demonstrate that NanoAmpli-Seq works equally well on the (now obsolete) 2D sequencing chemistry and the recently released 1D^2^ sequencing chemistry on the MinION device. While important limitations such as suboptimal reconstruction of community structure and an error rate of ∼0.5% remain, the proposed approach may be used for sequencing of long amplicons from complex microbial communities to assess community membership with cautious utilization of sequences from low-abundance OTUs due to likely lower sequence accuracy ranging from 99% to 99.5% accuracy.

## Results

### Experimental design and workflow

**Figure 1:**
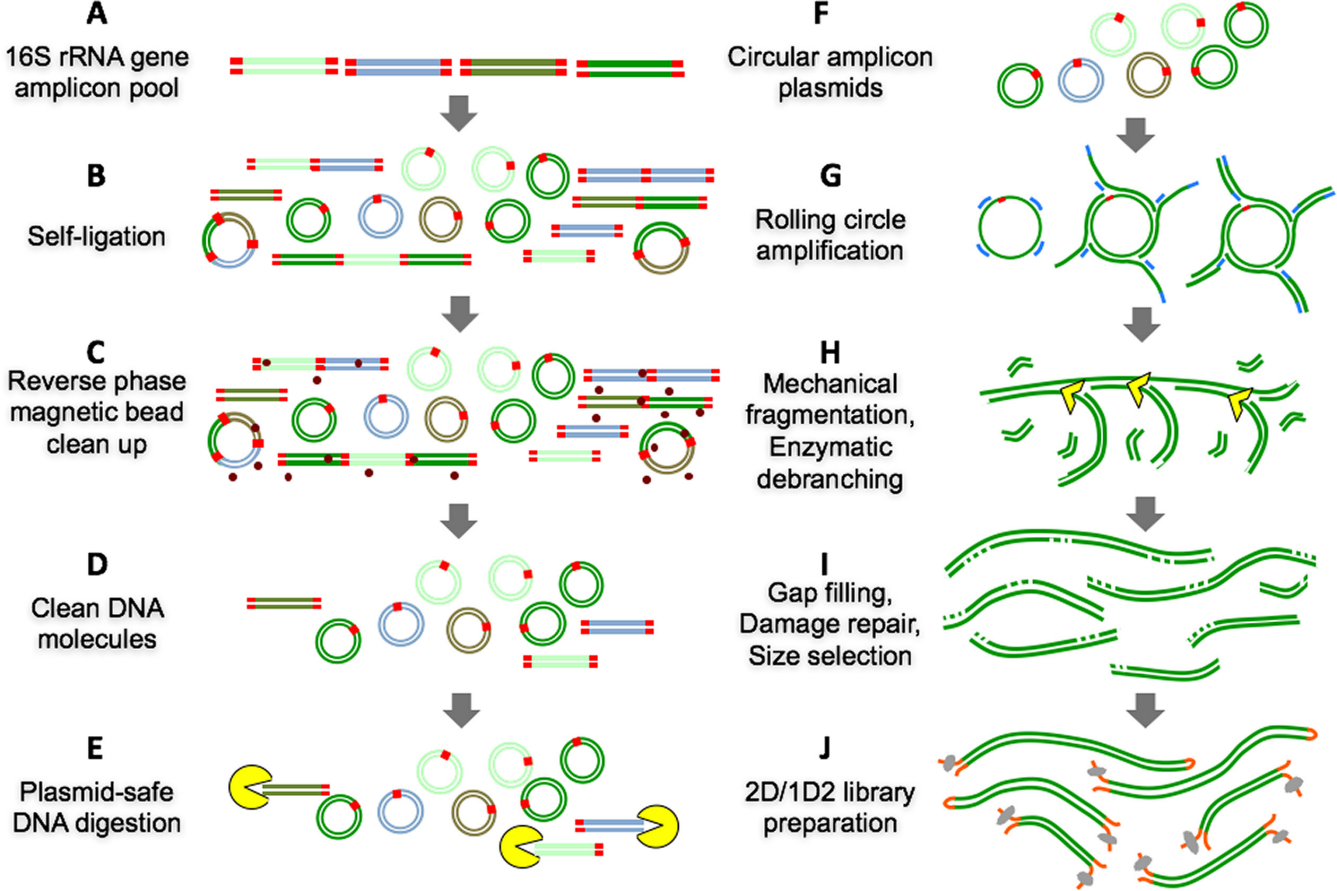
Overview of the sample preparation protocol for 16S rRNA gene amplicon pool preparation, plasmid-like structure construction, enzymatic debranching and mechanical fragmentation, and 2D and 1D^2^ library preparation including intermediate cleanup steps.

The NanoAmpli-Seq protocol was developed and validated using amplicon pools consisting of near full-length 16S rRNA gene of a single organism (*Listeria monocytogens*) or an equimolar amplicon pool of near full-length 16S rRNA genes from 10 organisms (Supplementary Table S1). The amplicon pools were generated by polymerase chain reaction (PCR) amplifying near full-length 16S rRNA genes from genomic DNA of the target organism(s) using primers and PCR reaction conditions as described in the Materials and Methods section. The respective amplicon pools were subsequently prepared for sequencing using the INC-Seq workflow as outlined in Fig. 1, with a few significant modifications. Briefly, the amplicon pools were self-ligated to form plasmid-like structures; this was followed by digestion with plasmid-safe DNAse to remove the remaining non-ligated linear amplicons. The DNA pool consisting of plasmid-like structures was subject to rolling circle amplification (RCA) using random hexamer-free protocol using a combination of primase/polymerase (PrimPol) and hi-fidelity Phi29 DNA polymerase [27]. The RCA product was subject to two rounds of T7 endonuclease I debranching and g-TUBE fragmentation followed by gap filling and DNA damage repair. Description of the protocol including reagent volumes and incubation conditions is provided in the Materials and Methods section, and a step-by-step protocol is provided in the Supplementary text. The prepared amplicon pools for both single-organism and 10-organism mock community samples were then subject to library preparation using the standard 2D (SQK-LSK208) (runs 1 and 2) and 1D^2^ (SQK-LSK308) (runs 3 and 4) kits using ONT specifications and sequenced on the MinION MK1b device followed by base calling using Albacore 1.2.4.

**Figure 2:**
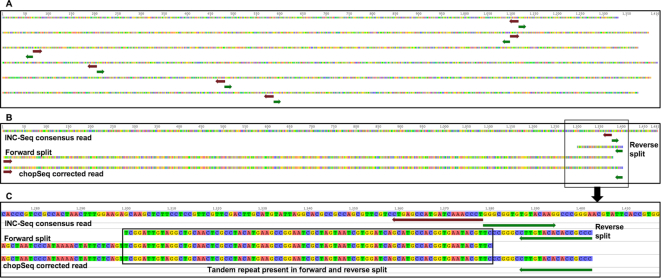
**(A)** Example of INC-Seq consensus reads showing the improper orientation with forward (maroon) and reverse (green) primers co-located and incorrectly oriented. **(B)** Manual splitting and re-orientation of the reads revealed the presence of tandem repeats in the forward and reverse splits that were identified and removed using chopSeq. **(C)** An expanded view of tandem repeat region in Fig. 2B.

Each resulting read consisted of multiple concatamerized physically linked amplicons from the one original 16S rRNA gene amplicon. The long concatemerized amplicon reads were subject to INC-Seq's anchor-based alignment and consensus error correction using three different alignment options (i.e., blastn, Graphmap, and partial order alignment [POA]) and followed by iteratively running PBDAGCON on the consensus for error correction (INC-Seq flag “iterative”). Reads with irregular segment length, unmappable anchors, and potentially chimeric molecules (i.e., concatemers from more than one original 16S rRNA gene amplicon) were removed during the generation of the INC-Seq consensus read. Manual inspection of INC-Seq consensus reads revealed that a vast majority had an incorrect orientation of primers (Fig. 2A). Specifically, the forward and reverse primers did not occur at the ends of the INC-Seq consensus reads but rather were co-located at varying positions along the length of each read. Efforts to manually split INC-Seq reads and re-orient the forward and reverse splits based on primer orientation revealed the presence of tandem repeats of nearly identical sequences, which affected efforts to merge the forward and reverse read splits (Fig. 2B, 2C).

To this end, we developed the chopSeq algorithm as part of the NanoAmpli-Seq workflow. The chopSeq algorithm uses pairwise2 open source library from Biopython package to identify user provided primers (forward “–f,” reverse “–r”) sequences including degenerate bases in the INC-Seq consensus reads. Primer detection is carried out in different orientations and primer match scores for each orientation are generated. Subsequently, primer sequences in the INC-Seq consensus read with the highest mean score are re-oriented, and any overhang is removed. Re-orienting reads using primer orientation resulted in the identification of insertions consisting of repeated sequence patterns, i.e., tandem repeats. These tandem repeats were identified using etandem algorithm from EMBOS open source software package [28], and various features of these repeats were delineated, i.e., tandem minimum repeat, tandem maximum repeat, and mismatch rate. The percent identity between tandem repeats is estimated iteratively measuring the sequence similarity between co-occurring segments using window size ranging from 10 bp to 350 bp with diminishing sequence similarity threshold with increasing window size. The sequence similarity threshold with increasing window size was applied as longer tandem repeats tend to have lower similarity to each other compared to shorter. After completing re-orientation of reads and the removal of tandem repeats, the forward and reverse splits are merged into a single read, and any read that does not match prescribed length threshold (i.e., 1300–1450 bp) is discarded. This process of primer identification and tandem repeat removal can also be visualized by turning on verbosity mode (flag = −v), and the results can be exported in fasta format.

**Figure 3:**
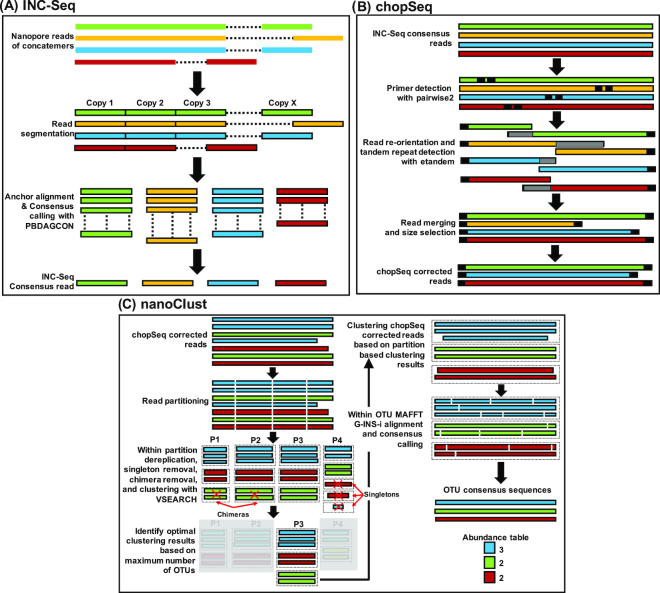
**(A)** Overview of the INC-Seq-based anchor alignment and iterative consensus calling using PBDAGCON. **(B)** INC-Seq consensus reads were subject to chopSeq-based read reorientation followed by tandem repeat removal and size selection to retain reads between 1300 and 1450bp. **(C)** chopSeq-corrected reads are subject to partitioning followed by VSEARCH-based binning to identify optimal binning results using partition that generates maximum number of OTUs (without singletons). MAFTT-G-INS-i was then used for sequence alignment of a subset of full-length reads from each OTU bin for the best-performing partition, and the alignment was used to create the OTU consensus read.

To enable fully reference-free analyses, we developed the nanoClust algorithm, which takes the fasta file of chopSeq-corrected reads as input and then performs read partitioning-based *de novo* clustering using VSEARCH [29] to delineate OTUs at a user-specified sequence similarity threshold (i.e., 97% in this study) followed by within-OTU read alignment and consensus calling for each OTU. The nanoClust algorithm is written in python, relies on Biopython packages, and was explicitly designed for *de novo* clustering because standard *de novo* clustering approaches such as VSEARCH [29] and the clustering approaches available in mothur [30, 31] vastly overestimated the richness of the mock community when using chopSeq-corrected reads (see details below). The nanoClust algorithm takes chopSeq-corrected reads in fasta format; splits the reads into partitions based on user-defined partition size; implements VSEARCH [29] for dereplication, chimera detection and removal in each partition, and clustering for each partition to identify the partition category with optimal (i.e., maximum) number of OTUs (not counting singleton OTUs); and discards singletons. Following this, nanoClust extracts read IDs for each OTU bin from the best-performing partition. The extracted read IDs for each OTU bin are then used to obtain full-length chopSeq-corrected reads; a subset of reads that fall within 10% of the average full-length read distribution within each OTU bin are aligned using Multiple Alignment using Fast Fourier Transform (MAFFT) [32, 33] with G-INS-i option, followed by consensus calling to obtain full-length representative sequence for each OTU. The entire data processing workflow is shown in Fig. 3.

### Modifications to the original INC-Seq protocol significantly reduces time required for amplicon concatemer pool preparation

While the proposed DNA preparation protocol is based on the previously developed INC-Seq approach [21], it contains multiple improvements that allow for faster and more efficient library preparation. These modifications include reduced incubation times for self-ligation step and plasmid-safe DNAse digestion process. More importantly, the current protocol utilizes Tth PrimPol [27] and Phi29 DNA polymerase enzymes for RCA that minimize the formation of unspecific products that may occur when using random hexamers. Similarly, the NanoAmpli-Seq protocol utilizes T7 endonuclease I enzyme for enzymatic debranching of RCA product combined with mechanical fragmentation step involving use of the g-TUBE. Thus, while our protocol increases the number of intermediate steps for sample preparation, by optimizing each step, it reduces the overall time required for sample DNA preparation to 6 hours (approximately 70% reduction compared to the original INC-Seq protocol). These improvements not only result in analyses of near full-length 16S rRNA gene (i.e., twice the amplicon size of the previously developed INC-Seq approach), but the combination of the improved protocol with appropriate data processing modifications resulted in significant increase in high-quality data post-processing.

### NanoAmpli-Seq data yield for 2D and 1D^2^ experiments

Runs 1, 2, 3, and 4 resulted in 29,420, 59,490, 142,233, and 301,432 raw records with post-base calling read lengths ranging from 5 bp to 43kbp and 5 bp to 234 kbp for 2D and 1D^2^ sequencing protocols (Supplementary Fig. S1). The pass reads to total raw reads ratio ranged from 28% for 2D to 7%–9% for the 1D^2^ experiments (Table 1). It is unclear if the low yield of pass reads, particularly for the 1D^2^ experiments, were due to the concatemerization process or DNA damage during enzymatic debranching and mechanical fragmentation that was unrepaired in the subsequent steps or due to base calling issues. All pass reads were subjected to INC-Seq processing to allow for consensus-based error correction using reads with a minimum of three concatemers per read (i.e., reads with less than three concatemers were excluded from any subsequent analyses) as compared to the six concatemer threshold used by Li et al. [21]. The number of concatemers per read passing INC-Seq threshold ranged from 3 to 21 and 3 to 42 for 2D and 1D^2^ data (Supplementary Fig. S1). The total number of reads passing the three concatemer threshold ranged from 36% to 75% of the base called reads depending on the experiment, sequencing protocol, and alignment approach during INC-Seq processing (Table 1). This was significantly higher than those reported by Li et al. [21], primarily due to the use of three compared to six concatemer threshold recommended previously.

**Table 1:** Summary of the total number of reads and median read lengths at each step of the data processing workflow for all experiments

	Number of reads				Median read length			
Protocol	2D	1D^2^	2D	1D^2^	2D	1D^2^	2D	1D^2^
Experiment	One organism	One organism	Ten organism	Ten organism	One organism	One organism	Ten organism	Ten organism
Run	1	4	2	3	1	4	2	3
Raw records	29,420	301,432	59,490	142,233	–	–	–	–
Pass reads	8,108	20,888	16,403	12,011	4,868	7,305	5,401	6,418
INC-Seq aligner	Blastn							
INC-Seq	3,911	7,618	7,011	9,081	1,394	1,433	1,396	1,413
chopSeq	3,911	7,618	7,011	9,081	1,383	1,387	1,377	1,374
chopSeq-size select	3,748	7,288	5,186	7,265	1,384	1,388	1,377	1,375
nanoClust	2,997	6,153	3,677	5,465	1,396	1,398	1,384	1,386
INC-Seq aligner	Graphmap							
INC-Seq	3,902	7,631	7,004	9,169	1,400	1,439	1,401	1,420
chopSeq	3,902	7,631	7,004	9,169	1,384	1,388	1,378	1,377
chopSeq-size select	3,765	7,399	5,190	7,496	1,384	1,389	1,377	1,377
nanoClust	2,981	6,179	4,141	5,490	1,397	1,396	1,384	1,386
INC-Seq aligner	POA							
INC-Seq	3,913	7,643	7,025	9,191	1,414	1,457	1,415	1,443
chopSeq	3,913	7,643	7,025	9,191	1,394	1,396	1,386	1,387
chopSeq-size select	3,779	7,088	5,076	6,954	1,394	1,396	1,385	1,386
nanoClust	3,184	5,993	3,916	5,622	1,398	1,389	1,384	1,386

### INC-Seq processed reads demonstrated incorrect read orientation and presence of tandem repeats

**Figure 4:**
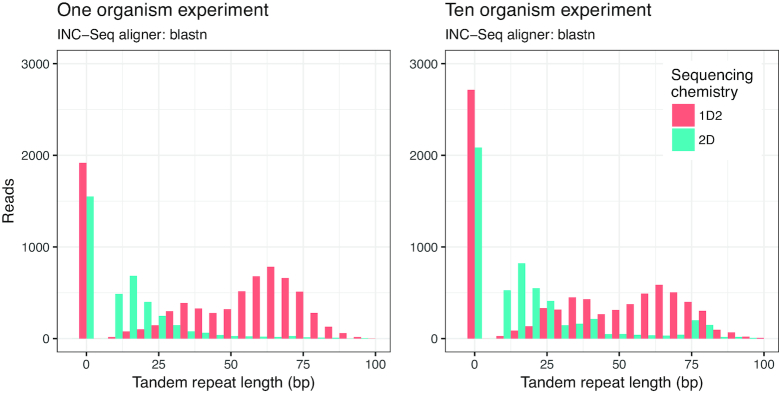
Histogram of tandem repeat length distribution of the INC-Seq consensus reads did not show any effect of the aligner used during the INC-Seq process but rather a marked effect of the sequencing chemistry. Results are only shown for INC-Seq consensus reads generated using blastn aligner.

While the median read lengths for post-INC-Seq were generally in the expected range (i.e., 1350–1450 bp) (Table 1) and similar for all three alignment methods used (Supplementary Fig. 2), manual inspection of the reads revealed several instances of incorrect read orientation (Fig. 2). The amplicon pool preparation protocol relies on RCA of plasmid-like structures constructed through self-ligation of linear amplicons followed by a combination of enzymatic debranching and mechanical fragmentation to generate linear molecules with multiple concatemers. Considering the fragmentation and debranching steps are not driven by sequence specificity, it would be reasonable to assume that the resulting linear amplicon is unlikely to have the correct orientation, i.e., 16S rRNA gene-specific forward and reverse primers do not flank the entire amplified region. Indeed, we found a vast majority of the 2D and 1D^2^ INC-Seq consensus reads were incorrectly oriented for the single organism sequencing runs, with forward and reverse primers not located at the ends of the reads. As a result, the reads were chopped at the primer sites and re-oriented to allow for the forward and reverse primers to be correctly oriented. However, during the process of read re-orientation, we also discovered the presence of inserts in the form of tandem repeats. Additional inspection of these inserts revealed that they were composed of multiple repetitive sequences, with the length of these inserts ranging from 10 bp to in excess of 1,500 bp (for rare cases), with median tandem repeat size ranging from 12 bp to 62 bp. The proportion of INC-Seq consensus reads with tandem repeats varied from 60% to 75% but did not reveal any significant effect of type of aligner used during INC-Seq consensus calling or the sequencing chemistry itself. Interestingly, however, the length distribution for the tandem repeats was strongly associated with the sequencing chemistry (Fig. 4).

Specifically, the 1D^2^ reads had longer tandem repeats as compared to the 2D reads and demonstrated a bimodal distribution of tandem repeat lengths as compared to the 2D data, which showed a unimodal tandem repeat length distribution (Fig. 4). While the template and complements in the 2D sequencing chemistry are physically linked by a hairpin adapter, they are not physically linked in the 1D^2^ sequencing chemistry; this could likely be the cause of differences in tandem repeat length distribution between 2D and 1D^2^ experiments.

### Read re-orientation and tandem repeat removal significantly improves sequence quality

**Figure 5:**
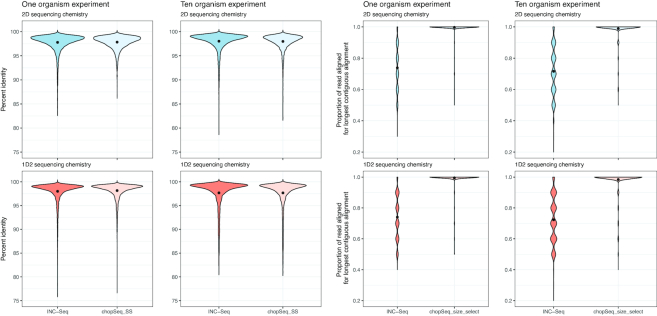
While the distribution of percent identities of INC-Seq and chopSeq processed reads (chopSeq_SS) to reference sequences was on average 97%–98%, variable lengths of the INC-Seq processed reads aligned to the reference sequences. In contrast, nearly the entire length of the chopSeq processed reads aligned to the reference sequence without affecting overall sequence similarity. Results are only shown for INC-Seq reads generated using blastn aligner.

Basic Local Alignment Search Tool n (BLASTn) analyses of INC-Seq reads against reference database composed of 16S rRNA gene sequences of 1 (run 1 and run 4) or 10 organisms (run 2 and run 3) revealed that a combination of incorrect read orientation and presence of tandem repeats significantly affected overall sequence quality. While the average sequence similarity between INC-Seq consensus reads and the reference sequences was 97 ± 0.37%, the portion of the INC-Seq consensus read demonstrating a contiguous alignment to the reference sequence varied significantly (Fig. 5); the remaining section of the read typically resulted in shorter secondary alignments with similar sequence similarity to that of the primary alignment. Post-chopSeq, the average proportion of the read aligning to the reference sequences increased from 73 ± 14.6% to 96 ± 2.3%. The additional step of discarding reads less than 1,300 bp and greater than 1,450 bp increased the average proportion of the read aligned to 98.4 ± 0.7%. The sequence similarity between chopSeq (97.5 ± 0.42%) and chopSeq followed by size selection (chopSeq_SS) (98 ± 0.23%) remained similar to or slightly better than INC-Seq processed read. This demonstrates that read reorientation and tandem repeat removal resulted in reconstruction of reads with a high level of similarity to the reference sequences (Fig. 5).

**Figure 6:**
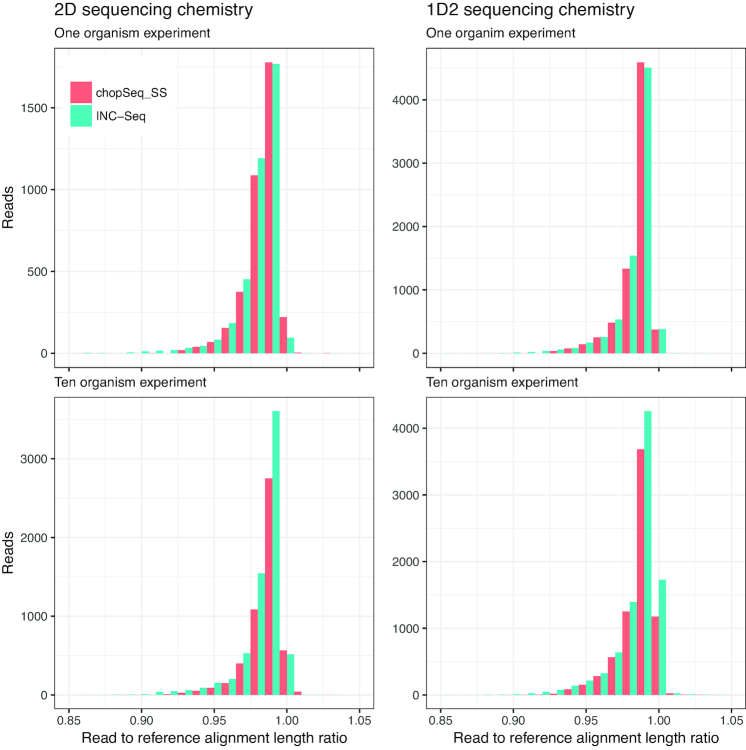
The ratio of the alignment length of INC-Seq and chopSeq-corrected reads to that of the corresponding reference sequences was consistently lower than 1, suggesting that deletions in the INC-Seq and chopSeq-corrected reads were the primary cause of dissimilarity with the reference sequences. Results are shown only for INC-Seq reads generated using blastn aligner.

Inspections of the read to reference alignment length ratio indicated that the primary source of sequence error for both INC-Seq and chopSeq-corrected reads originated from deletions; i.e., the majority of reads had a read to reference alignment ratio lower than 1. While deletions in reads were also strongly associated with sequence accuracy for post-chopSeq and size selected reads, a small proportion of chopSeq-corrected reads showed read to reference alignment ratios of greater than 1 (Fig. 6), suggesting that insertions were less prominent than deletions.

### *De novo* clustering of chopSeq-corrected sequences followed by within cluster consensus calling significantly enhances sequence accuracy

The overall sequence accuracy increased to an average of 98 ± 0.23% following chopSeq read correction and size selection, with 98.4 ± 0.7% of the read aligning to the reference (Fig. 5). However, approximately 5% and 10% of reads for the 2D and 1D^2^ runs, respectively, exhibited sequence accuracy of less than 95%, with some sequences aligning over less than 50% of the read length even after chopSeq correction and size selection. These poor-quality reads could not be selectively filtered out based on any commonly used quality filtering criteria (e.g., maximum homopolymers length, primer mismatches) and significantly affected clustering of reads into OTUs. For instance, VSEARCH-based clustering of full-length post-chopSeq and size selected reads (INC-Seq aligner: blastn) at a 97% sequence similarity threshold resulted in 817 (with 777 singletons) and 1,301 (with 1,238 singletons) for 2D and 1D^2^ data for single-organism experiments and 2,122 (with 1,742 singletons) and 2,725 (with 2,447 singletons) for 2D and 1D^2^ data for 10-organism experiments. We hypothesized that accrual of residual errors over the entire read length hampered the accuracy of the OTU clustering and that accurate clustering was more likely over shorter regions of the reads due to fewer absolute errors. To this end, we developed nanoClust, which utilizes partitioning of reads in user-defined lengths, followed by application of VSEARCH within each partition for dereplication, singleton removal, chimera detection and removal, and clustering at user-defined sequence similarity threshold (i.e., 97% sequence similarity in this study), followed by within-cluster sequence clustering and consensus calling.

**Figure 7:**
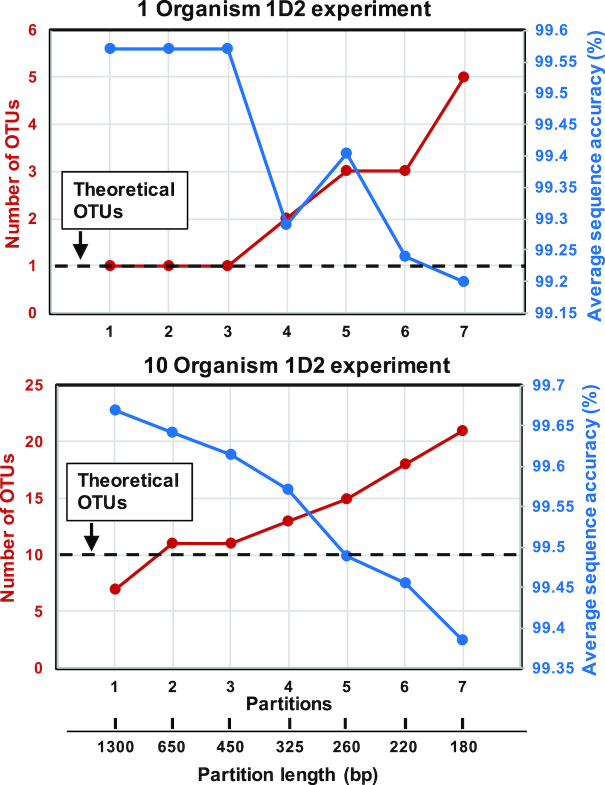
Increasing the number of partitions (and decreasing partition length) during nanoClust processing results in inflation in the number of OTUs observed and a decrease in overall sequence accuracy.

We tested the effect of the choice of partition length on the estimation of the richness of the mock communities (i.e., number of observed OTUs) and overall sequence accuracy post within-OTU MAFFT-G-INS-i alignment and consensus sequence construction. To this effect, we varied the number of partitions from one (i.e., partition length of 1,300 bp) to seven (i.e., partition length 180 bp). With increasing number of partitions (i.e., decreasing partition length), the number of OTUs being detected was significantly inflated above the theoretical threshold while at the same time the average sequence accuracy decreased (Fig. 7).

This was consistent for both the 1-organism and 10-organism experiments for both 1D^2^ and 2D experiments (Fig. 7). As the partition size increased (i.e., the number of partitions decreased), the number of OTUs decreased and the overall sequence accuracy increased. The highest average consensus sequence accuracy was observed with a single partition while the number of OTUs was lower than theoretical for the 10-organism experiment. Further, using a single partition approach also resulted in discarding a significant number of sequences that were deemed singletons prior to OTU clustering. Specifically, while three fewer OTUs were detected in the single partition approach, the total number of sequences retained post-clustering was 20% lower as compared to when a two- or three-partition approach was used. Considering the trade-off between sequencing depth (and the resultant impact on detection of lower-abundance OTUs) and the extent of deviation from the theoretical number of OTUs and overall sequence accuracy, we recommend using either the two- or three-partition approach, which results in similar outcomes on all three metrics.

**Figure 8:**
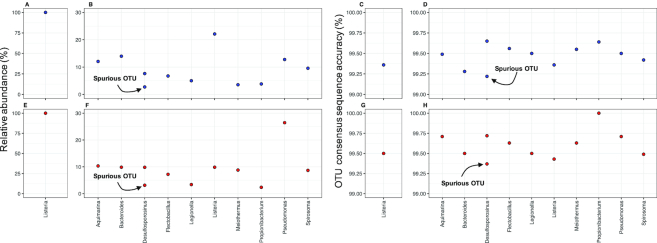
Relative abundance of OTUs for 1-organism **(A, E)** and 10-organism experiments **(B, F)** for 2D (blue data points) and 1D^2^ (red data points) experiments post-nanoClust when using blastn algorithm during INC-Seq. nanoClust clustering and consensus sequence generation resulted in few spurious OTUs and average similarity to the reference sequence of ∼99.5%. Results are shown for 1-organism **(C, G)** and 10- organism experiments **(D, H)** for 2D (blue data points) and 1D^2^ (red data points) experiments with the use of blastn during INC-Seq. The results were similar for Graphmap and POA alignment methods used during INC-Seq.

The nanoClust approach with three partitions was far superior to the direct clustering of full-length reads and resulted in an accurate determination of the number of OTUs, with one-two spurious OTUs (when using a three-concatemers threshold for INC-Seq) and no false negatives (Table 2) depending on the type of experiment and INC-Seq aligner used. MAFFT-G-INS-i alignment of 50 reads from each OTU resulted in consensus reads with the entire read aligned to the reference and average consensus read accuracy of 99.5% and accuracy values for individual OTUs ranging from 99.2% to 100% (Fig. 8). Nearly all errors in the nanoClust consensus reads originate from single base-pair deletions in a few homopolymers regions (homopolymers >4 bp) and no detectable insertions, with one-two mismatches associated with the spurious OTUs. While accurate OTU estimation allowed for single OTUs to be detected in the one-organism experiment, the overall community structure deviated from the theoretical community structure for the 10-organism experiments (Supplementary Fig. S4); thus, additional protocol optimization is essential to ensure that the levels of deviation from theoretical community structure do not exceed what may be seen from PCR biases [34]. Phylogenetic analyses of the consensus sequences demonstrated close placement of the OTU consensus sequences with their corresponding references, with excellent pairwise alignment between the two (Supplementary Fig. S3).

**Table 2:** Number of OTUs detected and consensus sequence accuracy for all experiments using the nanoClust for OTU clustering and consensus calling approach

	Number of OTUs				Average consensus accuracy (%)			
Protocol	2D	1D^2^	2D	1D^2^	2D	1D^2^	2D	1D^2^
Experiment	One organism	One organism	Ten organism	Ten organism	One organism	One organism	Ten organism	Ten organism
Run	1	4	2	3	1	4	2	3
Theoretical	1	1	10	10				
INC-Seq aligner	Blastn							
OTUs detected	1	1	11	11	99.36	99.5	99.47	99.61
Spurious OTUs	0	0	1	1	–	–	99.22	99.37
Non-Detect	0	0	0	0	–	–	–	–
INC-Seq aligner	Graphmap							
OTUs detected	1	1	11	11	99.43	99.43	99.44	99.61
Spurious OTUs	0	0	1	1	–	–	99.29	99.5
Non-Detect	0	0	0	0	–	–	–	–
INC-Seq aligner	POA							
OTUs detected	1	2	10	12	99.5	99.61	99.60	99.52
Spuriou**s**OTUs	0	1	0	2	–	99.57	–	98.67
Non-Detect	0	0	0	0	–	–	–	–

**Figure 9:**
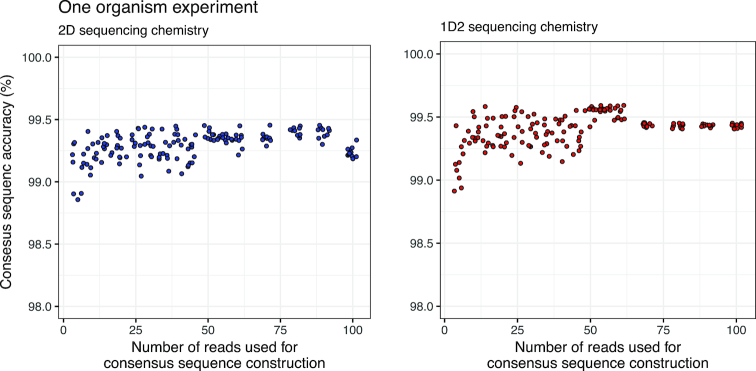
Consensus sequence accuracy plateaus with the use of 10–15 reads for MAFFT-G-INS-i alignment and consensus calling. However, with increasing number of reads used for consensus calling, the variability in consensus sequence accuracy from repeated sampling of data diminishes significantly for both 2D and 1D^2^ sequencing chemistry. Data are shown for one-organism experiment where the blastn aligner was used during INC-Seq.

The nanoClust implementation in this study included a specified threshold of a maximum of 50 reads per OTU to generate OTU consensus sequences. This was feasible because our study focuses on a single organism and even mock community of 10 organisms. Thus, the process of consensus construction was not limited by the number of reads that could be recruited. However, it would be critical to determine the potential for poor-quality consensus sequence due to fewer reads with an OTU in naturally derived mixed microbial communities. To this end, we varied the number of reads used for consensus sequence construction from 5 to 100 for 2D and 1D^2^ data from the one-organism experiment (Fig. 9).

The consensus sequence accuracy surpasses 99% with the use of more than five reads for consensus sequence construction and plateaus at approximately 10–15 reads. However, the variability in accuracy with repeated random sampling of data was much more pronounced when fewer than 50 reads were used for both 2D and 1D^2^ data. This suggests that consensus sequence accuracy is reliably high only for OTUs where a minimum of 50 reads are available for use in constructing the consensus sequence. This would have an impact on sequence quality of low-abundance OTUs.

### NanoAmpli-Seq-based improvements in sequence accuracy are not primarily associated with changes in nanopore sequencing chemistry

The INC-Seq study [21] utilized data generated from flow cells with R7 pores, while the present study used R9.4 and 9.5 pores with reported higher sequencing accuracy. Improvements in sequencing chemistry and base calling allowed us to reduce the concatemer threshold for INC-Seq from six to three, which significantly increased the amount of data used for analysis. The second significant improvement of the updated sequencing chemistry is the higher sequencing output. However, neither of these improvements result in improved data quality post-INC-Seq processing alone (Fig. 5). Thus, the chopSeq and nanoClust algorithms are critical for obtaining 99.5% sequence accuracy.

**Figure 10:**
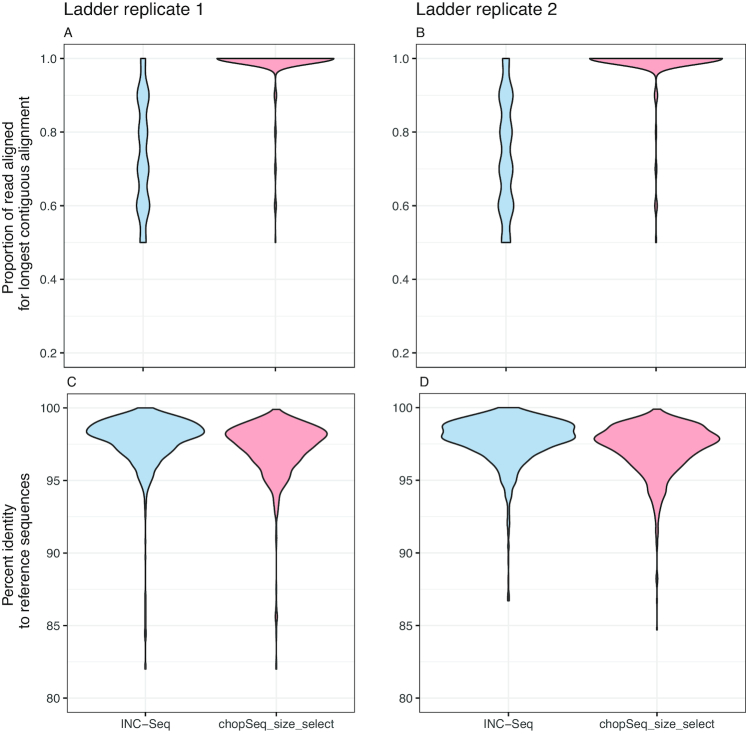
chopSeq-based read re-orientation and tandem repeat removal allowed for nearly the entire length of the read to be aligned to the reference sequences **(A, B)** while maintaining the median sequence accuracy of INC-Seq consensus reads to ∼97%–98%.

To demonstrate this, we re-processed the “Ladder replicate” data made available through the original INC-Seq study [21] using the NanoAmpli-Seq workflow. While re-analyzing R7 chemistry generated data from Li et al. [21], we detected tandem repeats and an incorrect primer orientation issue highlighted in this study. chopSeq was successfully able to remove tandem repeats and re-orient reads such that nearly the entire length of the read was now correctly aligned to the reference sequence for most of the reads. Thus, while INC-Seq reaches a median sequence accuracy of 97%–98% (as described in the Li et al. [21]), post-processing by chopSeq improves read quality through read re-orientation and tandem repeat removal (Fig. 10).

**Figure 11:**
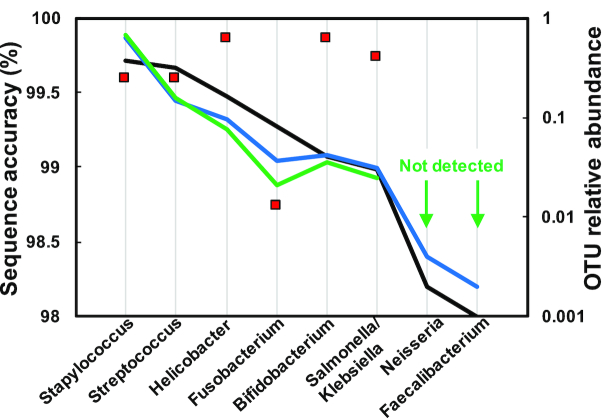
Sequence accuracy of detected OTUs post-nanoClust for combined ladder replicate was ∼99.5% (red squares). Two OTUs were not detected post-nanoClust processing (green line) due to their low abundance (<0.5%) in the constructed mock community (black line). The blue line shows the relative abundance as reported by Li et al.

Furthermore, nanoClust-based clustering and consensus calling results in an average sequence accuracy of 99.5% for the data generated by Li et al. [21]. The data generated by Li et al. [21] included the V3–V6 region of the 16S rRNA gene from 10-organism mock community with a staggered community structure including closely related organisms. This resulted in a theoretical number of eight OTUs at 97% sequence similarity. Specifically, *Staphylococcus aureus* and *Staphylococcus epidermis* clustered into a single OTU at 97% sequence similarity (their 16S rRNA gene V3–V6 hypervariable regions are 98.8% similar to each other) and *Klebsiella pneumoniae* and *Salmonella typhimurium* clustered into a single OTU at 97% sequence similarity (their 16S rRNA gene V3–V6 hypervariable regions are 97.6% similar to each other). Further, Li et al. [21] generated only 2,100 INC-Seq consensus reads combined for the two replicate sequencing runs. As a result, two of the low-abundance OTUs with a relative abundance of 0.2% (*Neisseria*) and 0.1% (*Faecalibacterium*) were not detected after processing with chopSeq and nanoClust. This non-detection of low-abundance OTUs is primarily a function of low sequencing depth rather than of the NanoAmpli-Seq workflow. Further, the sequence accuracy of most of the detected OTUs was in excess of 99.5%, while that of a single OTU (i.e., *Fusobacterium*) was 98.75% (Fig. 11). Thus, we conclude that incorporating chopSeq correction of INC-Seq consensus reads followed by nanoClust-based clustering and consensus calling was vital for improved sequence accuracy, irrespective of the changes in the sequencing chemistry.

## Discussion and Conclusions

The current study uses mock communities to develop and validate the NanoAmpli-Seq workflow for long amplicon sequencing on the nanopore sequencing platform. While this study focuses on the near full-length 16S rRNA gene, in principle the approach outlined by the NanoAmpli-Seq workflow should be amenable to amplicons generated from PCR amplification of any target gene irrespective of target gene length. While leveraging the previously described INC-Seq protocol, NanoAmpli-Seq adds several novel components that significantly enhance the amplicon sequencing workflow for the nanopore platform. The improvements over the previously described INC-Seq protocol involve modifications to both library preparation (i.e., PrimPol-based primer synthesis for RCA, debranching and fragmentation, shorter protocol length) and the data analyses. Specifically, we identify and fix the issues associated with incorrect read orientation and presence of tandem repeats in INC-Seq consensus reads, thus allowing for nearly the entire length of the chopSeq-corrected reads to be aligned to the reference with accuracies (97%–98%) similar to those described by Li et al. [21]. While the original INC-Seq protocol prescribed a concatemer threshold of six, we halved the concatemer threshold to three, thus, more than doubling the number of INC-Seq consensus reads available as a proportion of the base called reads. We could use a lower INC-Seq concatemer threshold due to both enhanced base calling and sequencing accuracy of the nanopore platform [35] and the ability to perform another round of alignment and consensus calling during the nanoClust step.

The correction of INC-Seq consensus reads using chopSeq did not allow for sequences with high enough quality for direct OTU clustering using VSEARCH. However, the read partitioning-based sequence clustering allowed for accurate determination of the number of OTUs in the mock community. Further, *de novo* sequence clustering using nanoClust provided the opportunity to significantly increase the number of sequences used for consensus calling. In this study, we used 50 reads for consensus calling (i.e., 150× coverage considering three concatemer threshold set for INC-Seq), which resulted in average sequence accuracy of 99.5%. The use of more than 50 reads for consensus calling in nanoClust did not improve sequence accuracy, while reducing the number of reads resulted in reduced precision. This threshold of 50 reads for both the 2D and 1D^2^ sequencing data suggests that the OTUs with fewer than 50 reads are likely to have sequence quality lower than those OTUs with greater than 50 reads. It should, however, be noted that using a 10-read threshold (i.e., 30× coverage [20] when including three concatemer threshold for INC-Seq) consistently allowed for sequence accuracy consistently higher than 99%; thus, sequence classification to species level using any of the current sequence classification approaches [36, 37] (i.e., RDP classifier) would be reliable even for lower-abundance OTUs.

While the NanoAmpli-Seq workflow represents a significant improvement in amplicon sequencing on the nanopore platform, some fundamental limitations remain. For instance, the NanoAmpli-Seq sequence accuracy is still lower than those reported for short amplicons [3] or those generated from the assembly of SSU rRNA from metagenomic sequencing on the Illumina platform [7, 9] and the full-length 16S rRNA sequencing on the PacBio platform using the approach described previously [17]. Our analysis shows that the sequence accuracy does not improve with more than 50 sequences used in the nanoClust-based consensus calling process. Nearly all of the errors in the OTU consensus sequences originate from single deletions at homopolymers regions, specifically for homopolymers greater than 4 bp. This homopolymer error issue on the nanopore platform is well known [14, 38] and is likely best resolved during the base calling process rather than subsequent data processing or by processing signal data (rather than base called data) all the way through clustering, followed by base calling as the final step. The second limitation of our approach is the low data yield at the base calling step, i.e., base called reads represents only a small portion (i.e., 7%–9% for 1D^2^ data) of the raw records. This data loss is significant and could potentially deter the widespread use of the nanopore platform for amplicon sequencing. While the precise cause of the low yield of pass reads post-base calling is unclear, the proportion of pass reads in our study is not significantly different from those reported elsewhere. One current option would be to directly work with 1D rather than 1D^2^ data. However, the maximum sequence accuracy of 1D reads post-INC-Seq consensus construction was only 94% and unsuitable for processing with chopSeq and nanoClust. The NanoAmpli-Seq workflow includes a *de novo* clustering step. As long as the sequence accuracy post-chopSeq is ∼97% (3–10 concatemers required), the binning process should provide for sufficient coverage for consensus-based sequence correction to accuracies in excess of 99%. The final limitation of our approach is that the nanoClust relies on generating consensus sequences from multiple DNA sequences and thus there is the likelihood of clustering and generating a multi-species consensus from closely related species, i.e., those within 97% sequence similarity to each other. While we did not find evidence for this “multispecies consensus sequence” while analyzing data from Li et al. [21], which included closely related organisms, this possibility cannot be ignored. Thus, we recommend that researchers refrain from depositing NanoAmpli-Seq processed sequences in publicly available references databases but rather utilize this approach for rapid screening of mixed microbial communities and limit the use of NanoAmpli-Seq processed data for within-study microbial community comparisons. Future improvement to avoid the likelihood of “multispecies consensus sequence” would be to utilize primers with bar codes consisting of random N bases (i.e., unique molecular tags), similar to that used by Karst et al. [10]. This could allow clustering of reads originating from the same original sequence using the unique molecular tags.

## Methods

### Mock community description and preparation

Two different mock communities were constructed for the experiments outlined in this study. First, a single-organism mock community was constructed by amplifying the near full-length of the 16S rRNA gene from genomic DNA of *Listeria monocytogens* using primers sets 8F (5′-AGRGTTTGATCMTGGCTCAG-3′) and 1387R (5′-GGGCGGWGTGTACAAG-3′), both with 5′ phosphorylated primers (Eurofins Genomics) [26]. Phosphorylated ends are essential for the subsequent self-ligation step. PCR reaction mix was prepared in 25  µL volumes with use of 12.5  µL of Q5 High-Fidelity 2X Master Mix (New England Biolabs Inc., M0492L), 0.8  µL of 10 pmol of each primer, 9.9  µL of nuclease-free water (Roche Ltd.), and 1 ng of bacterial DNA in total followed by PCR amplification as described previously [26]. PCR amplicons from replicate PCR reactions were combined and purified with use of HighPrep PCR magnetic beads (MagBio, AC-60 050) at 0.45× ratio. The 10-organism mock community was constructed from purified near full-length 16S rRNA amplicons of 10 organisms. Briefly, genomic DNA from 10 bacteria were obtained from DSMZ, Germany (Supplementary Table S1) and the aforementioned primers, PCR reaction mix, and thermocycling conditions were used to independently PCR amplify the near full-length 16S rRNA gene, followed by purification using HighPrep PCR magnetic beads as detailed above. The purified amplicons from each organism were quantified on the Qubit using dsDNA HS kit, normalized to 4 ng/µL, and combined to generate an amplicon pool consisting of an equimolar proportion of the 16S rRNA gene amplicons of the 10 organisms.

### DNA sequencing library preparation

To circularize the linear amplicons into plasmid-like structures, 5  µL of Blunt/TA Ligase Master Mix (New England Biolabs, M0367L) was added to 55  µL of amplicon pool at a concentration of 1  ng/µL and incubated for 10 minutes at 15°C then 10 minutes at room temperature (total time = 20 minutes). Not all linear amplicons self-ligate into plasmid-like structures, but some are likely to cause long chimeric linear amplicons. These long chimeric structures were removed using magnetic bead-based purification with the following modifications. HighPrep PCR magnetic beads were homogenized by vortexing followed by aliquoting 50  µL into a sterile 2-mL tube and placed on a magnetic rack for 3 minutes. A total of 25  µL of the supernatant was carefully removed using a sterile pipette to concentrate the beads to 2× its original concentration. The tube was removed from the magnetic rack and vortexed vigorously to resuspend the beads. This concentrated bead solution was used at a ratio of 0.35× to remove any amplicons greater than 2,000 bp in the post-ligation reaction mix. Briefly, the post-ligation product was mixed with concentrated bead solution at the 0.35× ratio by vortexing followed by incubation for 3 minutes at room temperature. The tube was placed on the magnetic rack to separate the beads from solution, followed by transferring of clear liquid containing DNA structures less than 2,000 bp into new sterile tubes. Sample containing short self-ligated molecules was subject to another round of concentration using standard magnetic beads at 0.5× ratios according to manufacturer's instructions and eluted in 15  µL of warm nuclease-free water. Concentrated and cleaned DNA pool consisting of plasmid-like structures and remaining linear amplicons was then processed with Plasmid-Safe ATP-Dependent DNase (Epicentre, E3101K) reagents to digest linear amplicons using the mini-prep protocol according to manufacturer's instructions and was followed by another round of cleanup with magnetic beads at 0.45× ratio as described before and then eluted in 15  µL of warm nuclease-free water.

The pool containing plasmid-like structures was subject to RCA with use of TruPrime RCA Kit (Sygnis, 390 100) random hexamer-free protocol. Samples were prepared in triplicate and processed according to manufacturer's protocol with all incubations performed in triplicate for 120–150 minutes depending on the assay efficiency. The progress of RCA was monitored by measuring the concentration of DNA using Qubit 2.0 Fluorometer at 90, 120, or 150 minute time points. The negative control sample, consisting of reagents without any circularized plasmid-like amplicons, was processed and analyzed concomitantly with the samples. The final concentration of the RCA product after 150 minutes of incubation was typically 70  ng/µL when using a starting DNA concentration of 0.35–0.4  ng/µL with no detectable unspecific product formation in the negative controls.

Replicate RCA products were combined (∼4.5  μg of DNA in total) and subject to de-branching and fragmentation of post-RCA molecules to remove hyperbranching structures generated during RCA. The RCA product was first treated with T7 endonuclease I enzyme (New England Biolabs, M0302S) by adding 2  µL of the reagent to the 65  µL of RCA product followed by vortexing and incubation as recommended by the manufacturer. Subsequently, the reaction mix was transferred into a g-TUBE (Covaris, 520 079) and centrifuged at 1,800 rpm for 4 minutes or until the entire reaction mix passed through the fragmentation hole. The g-TUBE was reversed, and the centrifugation process was repeated. Post-debranching and fragmentation, short fragments were removed using the modified bead-based cleanup step using concentrated bead solution (see above for concentration procedure). Concentrated beads were mixed with fragmented RCA product at a 0.35× ratio, vortexed for 15  seconds, and incubated at room temperature for 3 minutes, then placed on a magnetic rack until the beads separated and the supernatant was removed. The beads were subsequently washed with 70% freshly prepared ethanol according to the manufacturer's protocols. Size-selected amplicons bound to the beads were eluted in 41  µL of warm nuclease-free water. Preliminary experiments indicated that one round of de-branching did not completely resolve the hyperbranching structure, which was inferred based on poor sequencing yield likely caused by pore blocking by hyperbranched DNA. As a result, a second round of enzymatic de-branching using T7 endonuclease I was added, and the de-branched product was cleaned a second time using the bead-based cleanup step. Supplementary Fig. S4 shows example BioAnalyzer traces of the RCA product post-debranching/fragmentation and post-cleanup using magnetic bead-based protocol.

Finally, the de-branched RCA product was treated with NEBNext FFPE DNA Repair Mix (New England Biolabs, M6630S) for gap filling and DNA damage repair caused during g-TUBE fragmentation and T7 endonuclease I enzyme. All reagent components were combined with de-branched RCA product according to the manufacturer's recommendations and incubated at 12°C for 10 minutes then at 20°C for another 10 minutes. Post-incubation, the repaired RCA product was cleaned using standard magnetic beads at 0.5× ratio, washed with 70% ethanol, and eluted in 46  µL of warm nuclease-free water. The concentration of the DNA product was measured using Qubit and was approximately 20–25  ng/µL, with a total yield of ∼1,000 ng of DNA with product size typically ranging from 1,500 bp to 20,000 bp. A total of 45  µL DNA pool of concatemerized amplicons was prepared for sequencing using the standard 2D and 1D^2^ library preparation protocol by ONT (SQK-LSK208, SQK-LSK308) according to the manufacturer's specifications to obtain pre-sequencing mix. Moreover, the final concentration for prepared libraries was determined using dsDNA HS kit on the Qubit instrument. A detailed step-by-step protocol is provided in the Supplementary text.

### DNA sequencing

The MinION MkIB was connected to Windows personal computer compatible with ONT requirements. R9.4 (FLO-MIN106) and R9.5 (FLO-MIN107) flow cells were placed onto the MinION Mk1B (ONT). Platform quality control was performed using MinKNOW software (v1.4.2 for 2D and v1.6.11 for 1D^2^ libraries). Only flow cells containing more than 1,100 active pores were used in this study. Each flow cell was primed twice according to ONT specifications using priming buffer consisting of equal parts of running buffer (RBF1) and nuclease-free water with 10-minutes breaks between subsequent primes. The loading mix was prepared with a 12  μL pre-sequencing mix, 75  μL of RBF1, and 63  μL of nuclease-free water. Loading mix was sequenced with MinKNOW settings appropriate for 2D or 1D^2^ options and standard 48 hour processing time for every run. Albacore 1.2.4 was used to convert raw signals into HDF5 file format using switch options FLO-MIN106 and SQK-LSK208 for 2D data and FLO-MIN107 and SQK-LSK308 for 1D^2^ data.

### Data processing

HDF5 raw signals from each sequencing run were converted to FASTQ format using Fast5-to-Fastq [39] and then from FASTQ to FASTA with seqtk [40]. The resultant data were subject to INC-Seq [21] processing using blastn, Graphmap, and POA aligners with concatemer threshold of 3 with the iterative flag for consensus error correction using PBDAGCON. INC-Seq consensus reads were subject to chopSeq by specifying forward and reverse primer sequences and upper (1,450 bp) and lower (1,300 bp) read size thresholds for size selection (chopSeq_SS). The chopSeq-processed reads were subsequently processed using nanoClust with partition size limits (flag “-s”) of 0,450,451,900,901,1300, which splits the reads into three partitions of 450, 450, and 400 bp, respectively, prior to further processing. VSEARCH was used for chimera removal (using uchime) followed by clustering of reads in each partition at a hard-coded sequence similarity threshold of 97%. For the optimal binning results, nanoClust calculates local average length of the reads present for each OTU generated with VSEARCH. This is followed by MAFFT-G-INS-i multiple sequence alignment followed by majority consensus calling of the first 50 reads present at the size variability threshold (+/−10%). Finally nanoClust outputs an OTU table with reads corresponding to each OTU after discarding singletons.

Fasta files from all stages (i.e., raw, INC-Seq, chopSeq, chopSeq_SS, and OTU consensus sequence) were analyzed for read lengths in R [41]. The number of concatemers on each raw read was estimated by dividing the length of each raw read with the length of its corresponding INC-Seq consensus read. At each stage of processing, the reads were aligned to reference dataset using blastn, and only the match with the highest bit score was considered. The ratio of read to reference alignment at appropriate points (as discussed above) was estimated based on blastn results. The percent identity from the blastn results was used to measure consensus sequence accuracy for the nanoClust output. All figures for the manuscript were generated in R using packages “ggplot2” [42], “gridExtra” [43], and “cowplot” [44], as appropriate. Neighbor-joining tree construction (Supplementary Figs. S3 and S4) was performed after muscle alignment [45] (default parameters) and using Jukes-Cantor model was constructed in Geneious (version 8) using 100 bootstraps.

## Availability of source code and requirements

Project name: NanoAmpli-Seq

Project home page: https://github.com/umerijaz/nanopore

Operating system: Linux

Programming language: Python

License: MIT License

RRID:SCR_016710

## Availability of supporting data

All data are available in the European Nucleotide Archive under primary accession number PRJEB21005. Intermediate and final fasta files for amplicon sequencing processing workflow are included in the *GigaScience* GigaDB repository [46].

## Additional files

Supplementary material_R2.pdf

## Abbreviations

BLAST: Basic Local Alignment Search Tool; INC-Seq: intramolecular-ligated nanopore consensus sequencing; MAFFT: Multiple Alignment using Fast Fourier Transform; ONT: Oxford Nanopore Technologies; OTU: operational taxonomic unit; PacBio: Pacific Bioscience; PCR: polymerase chain reaction; POA: partial order alignment; RBF: running buffer; RCA: rolling circle amplification; SMRT: single-molecule real-time sequencing; SSU: small subunit.

## Competing Interests

No competing interest to declare.

## Funding

This research was supported by Engineering and Physical Sciences Research Council (EPSRC) (award EP/M016811/1). S.T.C. is supported by the EPSRC Doctoral Training Center at University of Glasgow. U.Z.I. is supported by a Natural Environment Research Council Fellowship (NE/L011956/1).

## Author contribution

S.T.C., U.Z.I., and A.J.P. designed and developed the experiments. S.T.C. performed the experiments. U.Z.I. wrote the code for chopSeq and nanoClust. S.T.C., U.Z.I., and A.J.P. analyzed the data and contributed equally to writing the manuscript.

## Supplementary Material

giga-d-18-00251_original_submission.pdf

giga-d-18-00251_revision_1.pdf

giga-d-18-00251_revision_2.pdf

response_to_reviewer_comments_original_submission.pdf

response_to_reviewer_comments_revision_1.pdf

reviewer_1_report_(original_submission) -- Chenhao Li 8/7/2018 Reviewed

reviewer_2_report_(original_submission) -- Amrita Srivathsan 8/16/2018 Reviewed

reviewer_3_report_(original_submission) -- Lee Kerkhof 8/18/2018 Reviewed

Supplemental Files
