# Supplementary material for: NanoAmpli-Seq: a workflow for amplicon sequencing for mixed microbial communities on the nanopore sequencing platform
Source: Gigascience. 2018 Nov 23;7(12):giy140. doi: 10.1093/gigascience/giy140 (PMC6298384; doi:10.1093/gigascience/giy140)

NanoAmpli-Seq: A workflow for amplicon sequencing for mixed microbial communities on the nanopore sequencing platform.

Szymon T Calus, Umer Z Ijaz, Ameet J Pinto

Protocol for sample preparation for NanoAmpli-Seq workflow.

Consumables required:

1. Sterile Filtered pipette tips from any vendor
2. 0.2 ml PCR grade tubes from any vendor
3. Wide Bore Filtered pipette tips from any vendor
4. Qubit dsDNA HS Assay kit. Vendor: ThermoFisher Scientific. Catalog number: Q32851.
5. Primers for PCR amplification of 16S rRNA gene with phosphorylated 5' ends can be ordered from any provider:
    a. Forward primer: 8F: [PHO] AGRGTTTGATCMTGGCTCAG
    b. Reverse primer: 1387R: [PHO] GGGCGGWGTGTACAAGRC
6. The master mix for PCR amplification: Q5® High-Fidelity 2X Master Mix. New England Biolabs, Inc. Catalog number: M0492S
7. Nuclease-free Water. Vendor: New England Biolabs, Inc. Catalog number: B1500S
8. HighPrep™ PCR paramagnetic bead solution. Vendor: MAGBIO. Catalog number: AC-60050
9. 70% ethanol (prepared from denatured ethanol).
10. Magnetic stand for 1.5 ml tubes. MagStrip Magnet Stand 10. Vendor: MAGBIO. Catalog number: MBMS-10
11. Blunt/TA Ligase Master Mix. Vendor: New England Biolabs, Inc. Catalog number: M0367S
12. DNA LoBind Tubes, 1.5 ml. Vendor: Eppendorf. Catalog number: 003018078
13. Plasmid-Safe™ ATP-Dependent DNAse. Vendor: Epicentre. Catalog number: E3101K.
14. TruePrime™ RCA kit. Vendor: Expedeon. Catalog number: 390100
15. T7 endonuclease I. Vendor: New England Biolabs, Inc. Catalog number: M0302S.
16. g-TUBE. Vendor: Covaris. Catalog number: 010145
17. NEBNext® FFPE DNA Repair Mix. Vendor: New England Biolabs Inc. Catalog number: M6630S
18. Ligation Sequencing Kit 1D. Catalog number: SQK-LSK108 and/or Ligation Sequencing Kit $1D^2$. Catalog number: SQK-LSK308. Vendor: Oxford Nanopore Technologies
19. Flow Cell (R9.4) and/or Flow Cell (R9.5). Vendor: Oxford Nanopore Technologies

Equipment required:

1. PCR thermocycler from any vendor
2. PCR hood
3. Thermal mixer with appropriate blocks from any vendor
4. Pipettes from varying volumes range from any vendor
5. Centrifuge for 2 ml and 0.2 ml tubes from any vendor
6. MinION™ Mk1b device and compatible personal computer

Step 1: PCR amplification of 16S rRNA gene.

1. Combine the following components using volumes below or as appropriate for your experiment in a PCR tube.
   a. Q5® High-Fidelity 2X Master Mix: 12.5 µl
   b. Forward primer (10 pmol): 0.8 µl
   c. Reverse primer: (10 pmol): 0.8 µl
   d. Template DNA: 1 µl
   e. Nuclease-free water: 9.9 µl.

2. Amplify PCR reaction mix at the following PCR conditions:

Segment	Temp (°C)	Time (sec)	Cycles
Initial denaturation	98	00:30	1
Denaturation	98	00:05	20
Annealing	59	00:10	
Extension	72	00:35	
Final extension	72	02:00	1
Hold	8	00:00	1

Step 2: PCR product clean up.

1. Follow vendor instructions (MAGBIO) to clean PCR product at 0.45x bead ratio as described at the following URL: http://www.magbiogenomics.com/image/data/Literature/Protocols/HighPrep%20PCR%20Protocol.pdf

Step 3: PCR product concentration estimation.

1. Follow vendor instructions (ThermoFisher) to determine the concentration of cleaned PCR product using Qubit™ dsDNA HS Assay kit as described at the following URL: https://assets.thermofisher.com/TFS-Assets/LSG/manuals/Qubit_dsDNA_HS_Assay_UG.pdf

Step 4: Self-ligation for the formation of plasmid-like structure.

1. Dilute PCR product from step 2 to 2-3 ng/µl using nuclease-free water
2. Mix 55 µl of diluted PCR product with 5 µl of Blunt/TA Ligase Master Mix in 0.2 ml PCR grade tubes
3. Gently mix by flicking the tube a few times
4. Centrifuge tube for 10 seconds
5. Incubate for 10 min at 15°C in a PCR thermocycler
6. Gently mix by flicking the tube a few times
7. Centrifuge tube for 10 seconds
8. Incubate for another 10 min at room temperature.

Step 5: Reverse phase cleanup.

1. Vortex HighPrep™ PCR paramagnetic bead solution
2. Transfer 50 µl of bead solution into clean DNA LoBind 1.5 ml tube

3. Place the tube on a magnetic rack until beads separate from the liquid
4. While the tube is on the magnetic rack, remove 25 µl of liquid from the tube using sterile pipette tip, taking care not to disturb the beads
5. Remove the tube from magnetic rack and gently vortex to resuspend the beads.
6. Add the self-ligation mix from step 4 to concentrated beads at a ratio of 0.35x bead ratio.
7. Incubate the mixture for 3 minutes at room temperature.
8. Place tube on a magnetic rack
9. Allow the beads to separate from the liquid. The beads contain long linear amplicons (potentially chimeric amplicons) while the liquid contains short linear amplicon and plasmid-like structures. Carefully remove the clear liquid from the tube and move it to step 6.

Step 6: Plasmid and short amplicon clean-up.

1. Follow vendor instructions (MAGBIO) to clean plasmid and short amplicon mix at 0.45x bead ratio as described at the following URL: http://www.magbiogenomics.com/image/data/Literature/Protocols/HighPrep%20PCR%20Protocol.pdf

Step 7: Removal of linear molecules from plasmid mix.

1. Combine 42 µl of clean product from step 6 (eluted in nuclease-free water) with 2 µl of 25 mM ATP solution, 5 µl of 10X reaction buffer and 1 µl of Plasmid-Safe DNASe in 0.2 ml PCR grade tube. The last three ingredients are provided with the Plasmid-Safe™ ATP-Dependent DNAse kit.
2. Incubate at 37°C for 15 minutes in a PCR thermocycler
3. Clean product as described in Step 2
4. Determine the concentration of DNA in the cleaned product as described in Step 3.

Step 8: Rolling Circle Amplification (RCA).

Perform RCA in triplicate for each sample and include negative controls using nuclease-free water instead of cleaned product from step 7. Below are reaction conditions and volumes for a single RCA reaction:

1. Combine 2.5 µl of cleaned product from step 7 with 2.5 µl of Buffer D (provided with TruePrime™ RCA kit) in 0.2 ml PCR grade tube and incubate at room temperature for 3-5 minutes
2. While the sample is being incubated, prepare the amplification mix consisting of 9.3 µl of nuclease-free water, 2.5 µl of reaction buffer, 2.5 µl of dNTPs, 2.5 µl of Enzyme 1 and 2.5 µl of Enzyme 2. All ingredients are included in the TruePrime™ RCA kit
3. After 10 minutes, add 2.5 µl of Buffer N (provided with TruePrime™ RCA kit) and prepared amplification mix to the tube
4. Mix by pipetting and incubate tube at 30°C for 150 minutes.
5. Follow Step 3 to determine the concentration of DNA in samples and negative controls.

Step 9: Enzymatic de-branching.

1. Combine triplicate reactions from step 8 into a single 0.2 ml PCR grade tube
2. Mix thoroughly to prepare single RCA product per sample

3. Combine 65 µl of RCA product with 2 µl of T7 endonuclease I and incubate at room temperature for 5 minutes.

Step 10: Mechanical fragmentation.

1. Transfer enzymatically de-branched RCA product into g-TUBE using wide bore pipette tips
2. Centrifuge at 1800 rpm for 4 minutes or until entire reaction mix passes through the fragmentation hole
3. Reverse the g-TUBE and centrifuge again at 1800 rpm for 4 minutes or until entire reaction mix passes through the fragmentation hole
4. Clean product with as described in Step 5, with the exception that the clear liquid after bead separation is discarded and bead-bound DNA is eluted according to vendor outlined protocol
5. Repeat step 9 (i.e. Enzymatic de-branching) on the g-TUBE fragmented product.
6. Clean product as described in Step 5, with the exception that the clear liquid after bead separation is discarded and bead-bound DNA is eluted according to vendor outlined protocol.

Step 11: DNA Damage repair.

1. Combine 53.5 µl of product from Step 10 with 6.5 µl of FFPE DNA Repair Buffer and 2 µl of NEBNext FFPE Repair mix in a 0.2 ml tube
2. Incubate at 20°C for 15 minutes
3. Clean product as described in Step 2
4. Quantify DNA concentration as described in Step 3.

Step 12: **DNA library preparation and nanopore sequencing.**

1. Prepare 2D or $1D^2$ libraries for nanopore sequencing according to the protocols described for the SQK-LSK108 or SQK-LSK308 kits by Oxford Nanopore Technologies.
2. Primer appropriate flow cell using protocols outlined by Oxford Nanopore Technologies.
3. Finally, sequence the prepare libraries on appropriate flow cells as outlined by Oxford Nanopore Technologies.

Table S1: Names and DSM catalog numbers of bacteria used to construct mock communities for the single organism and ten organism experiments and their corresponding accession numbers are shown below. The 16S rRNA genes were extracted from genome assemblies using RNAmmer[1] for bacteria for use in estimation of sequencing accuracy. Where genome assemblies were unavailable, the 16S rRNA gene sequence in Genbank was utilized.

Organism name	DSM catalog number	Genbank assembly accession number	16S rRNA gene accession number
One organism experiment			
Listeria monocytogenes	19094	HE999705.1	
Ten organism experiment			
Aquimarina intermedia	17527	-	AM113977
Bacteroides vulgatus	1447	CP000139	-
Desulfosporosinus orientis	765	CP003108	-
Flectobacillus major	103	ATXY00000000	-
Legionella pneumophila	7513	AE017354	-
Listeria monocytogenes	19094	HE999705.1	-
Meiothermus ruber	1279	CP001743	-
Propionibacterium acnes	16379	AE017283	-
Pseudomonas aeruginosa	1128	NC_009656	-
Spirosoma linguale	74	CP001769	-

1 Lagesen, K. *et al.* RNAmmer: consistent and rapid annotation of ribosomal RNA genes. *Nucleic Acids Research* **35**, 3100-3108, (2007).

Figure S1: Violin plots showing the read length distribution of raw data (i.e., post base calling with Albacore 1.2.4), and the number of concatemers on each base called read estimated using read lengths from INC-Seq processing using the "blastn" alignment approach for all four experiments involving both 2D and $1D^2$ chemistry.

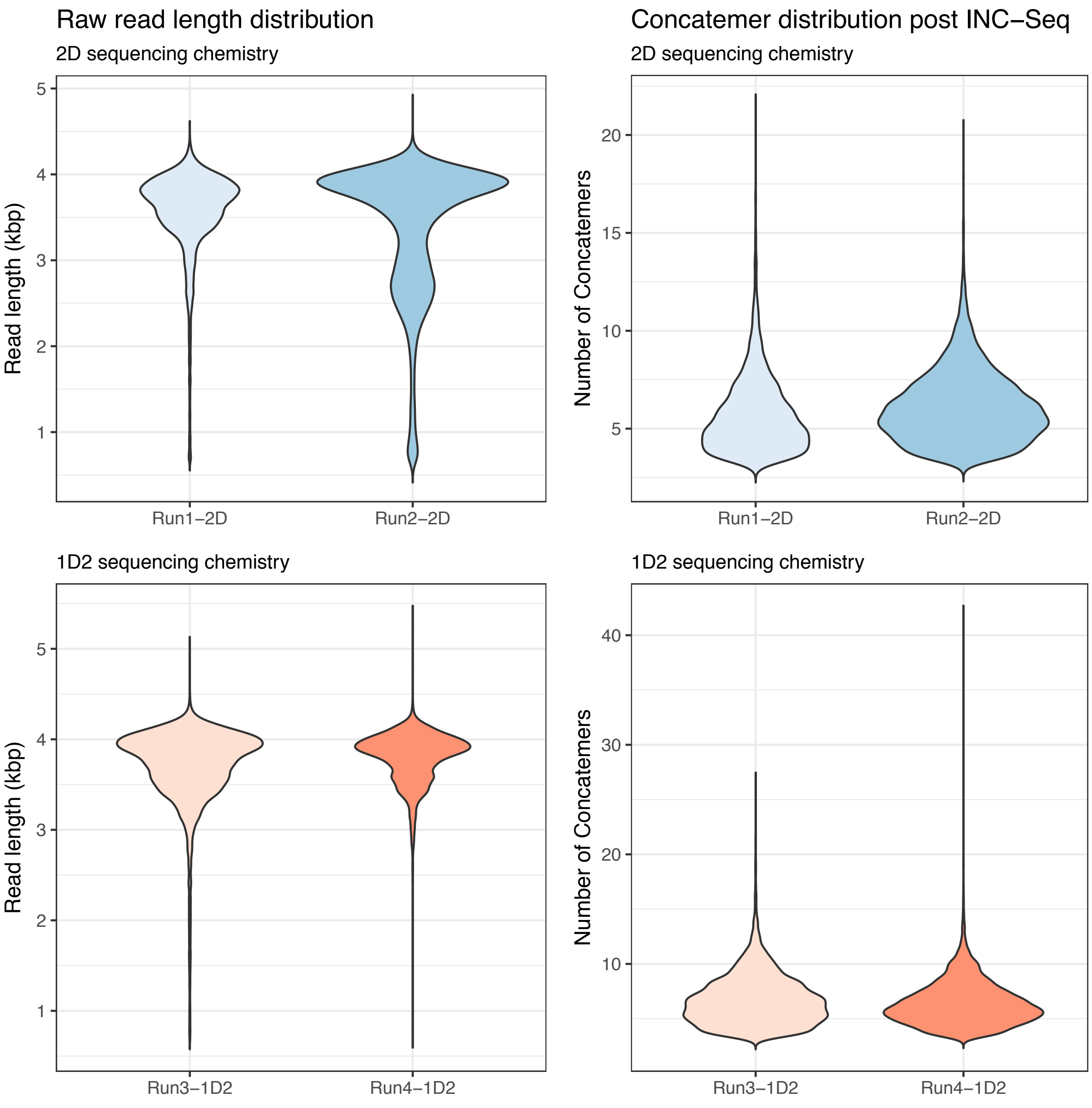

Figure S2: Violin plot of read length distributions for INC-Seq processed reads, chopSeq corrected reads after tandem repeat removal, and post-size-selection of chopSeq corrected reads and size selected reads (chopSeq_SS) for one organism experiments using both 2D and $1D^2$ sequencing chemistry.

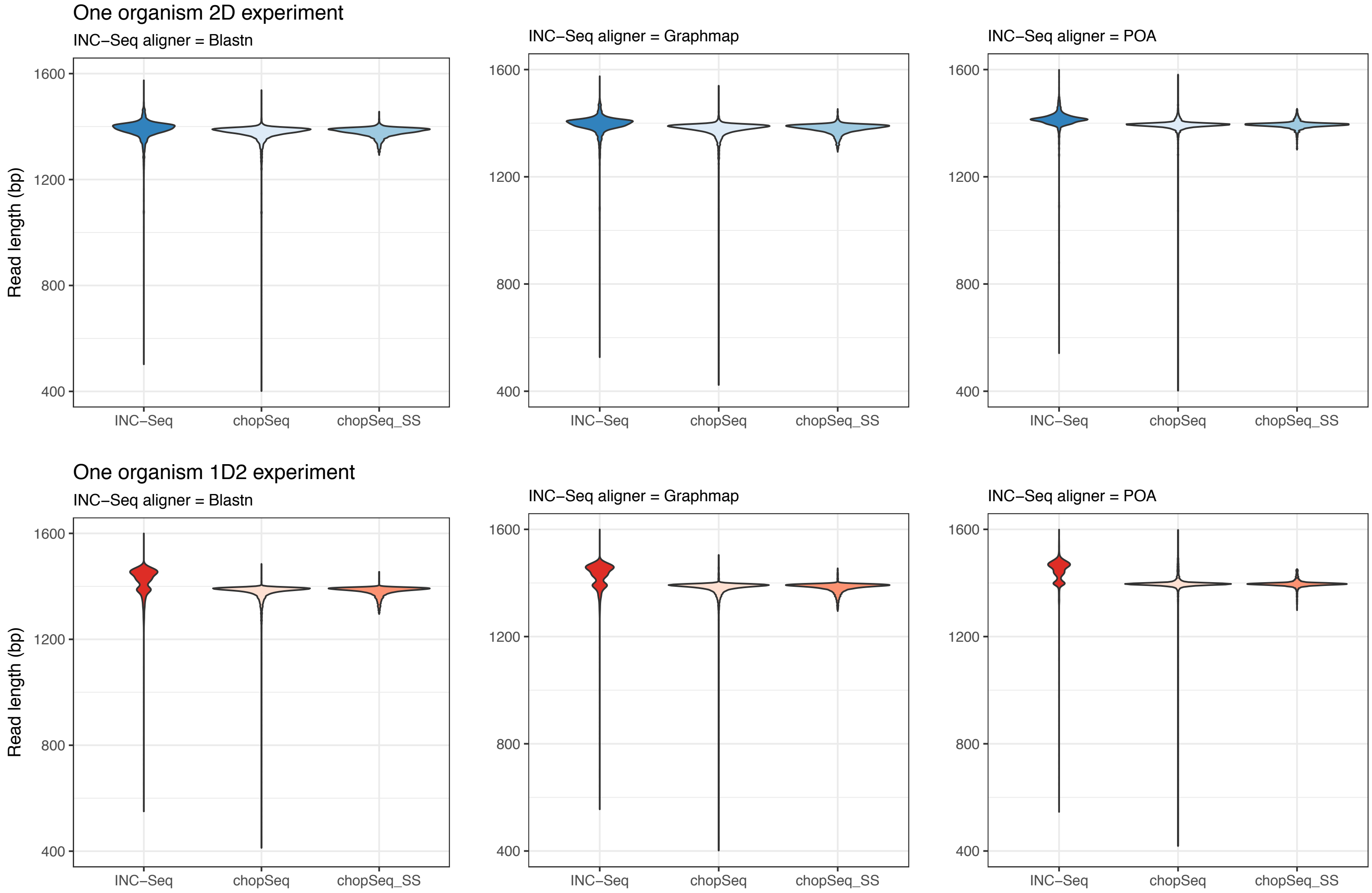

Figure S3: Consensus sequences from OTUs from Run 3 ($1D^2$ sequencing chemistry, INC-Seq aligner: blastn) were combined with reference sequences and aligned using muscle (default parameters) and Neighbor-Joining tree using Jukes-Cantor model was constructed in Geneious (version 8) using 100 bootstraps. Reference sequences are labelled in green, and OTUs are labelled in red.

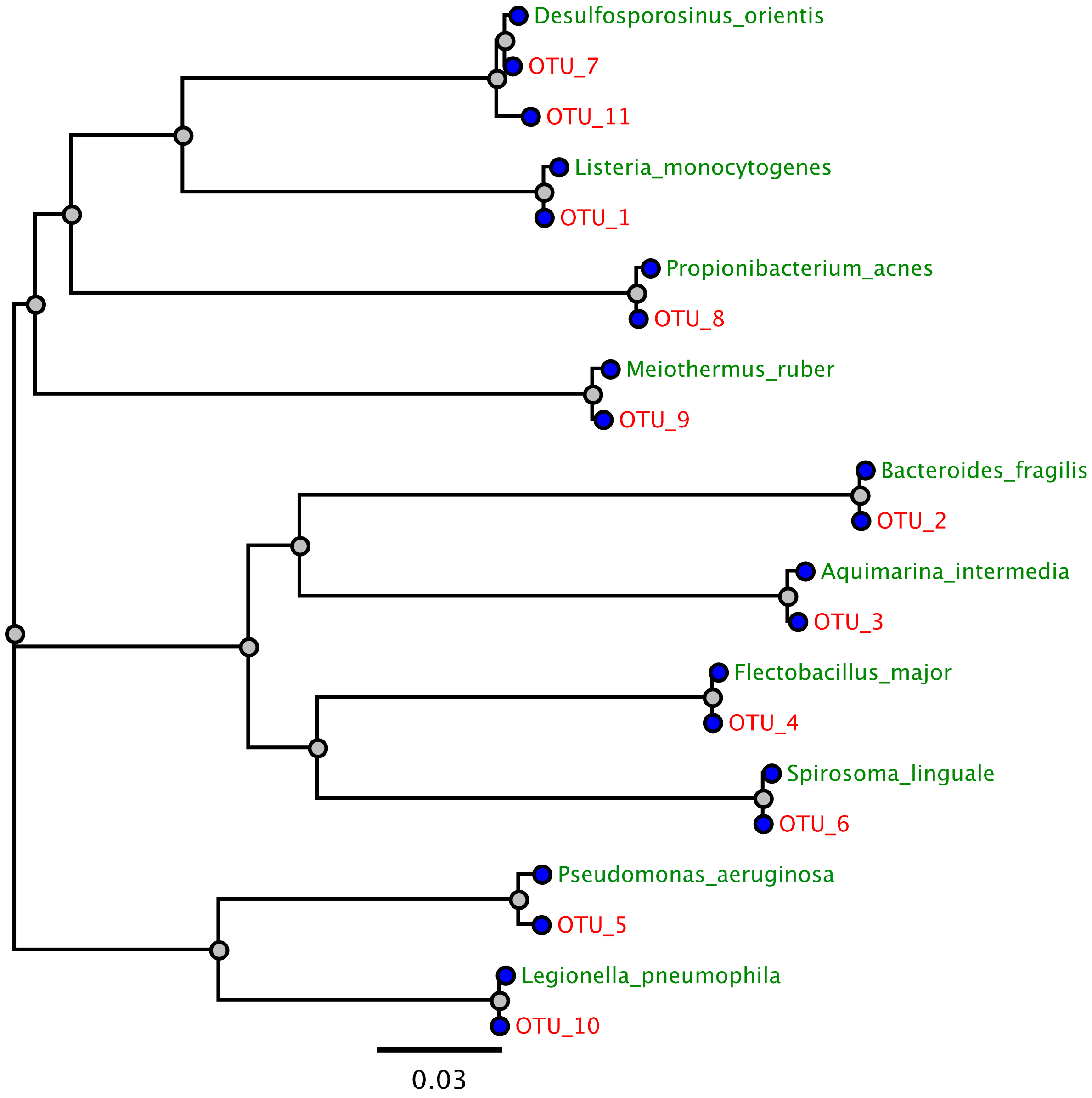

Figure S4: Example Bioanalyzer traces of de-branched product pre- and post-cleanup. The post-cleanup product was then treated for gap filling and DNA damage repair prior to being used for library preparation of sequencing according to ONT protocol for 2D and $1D^2$ chemistries.

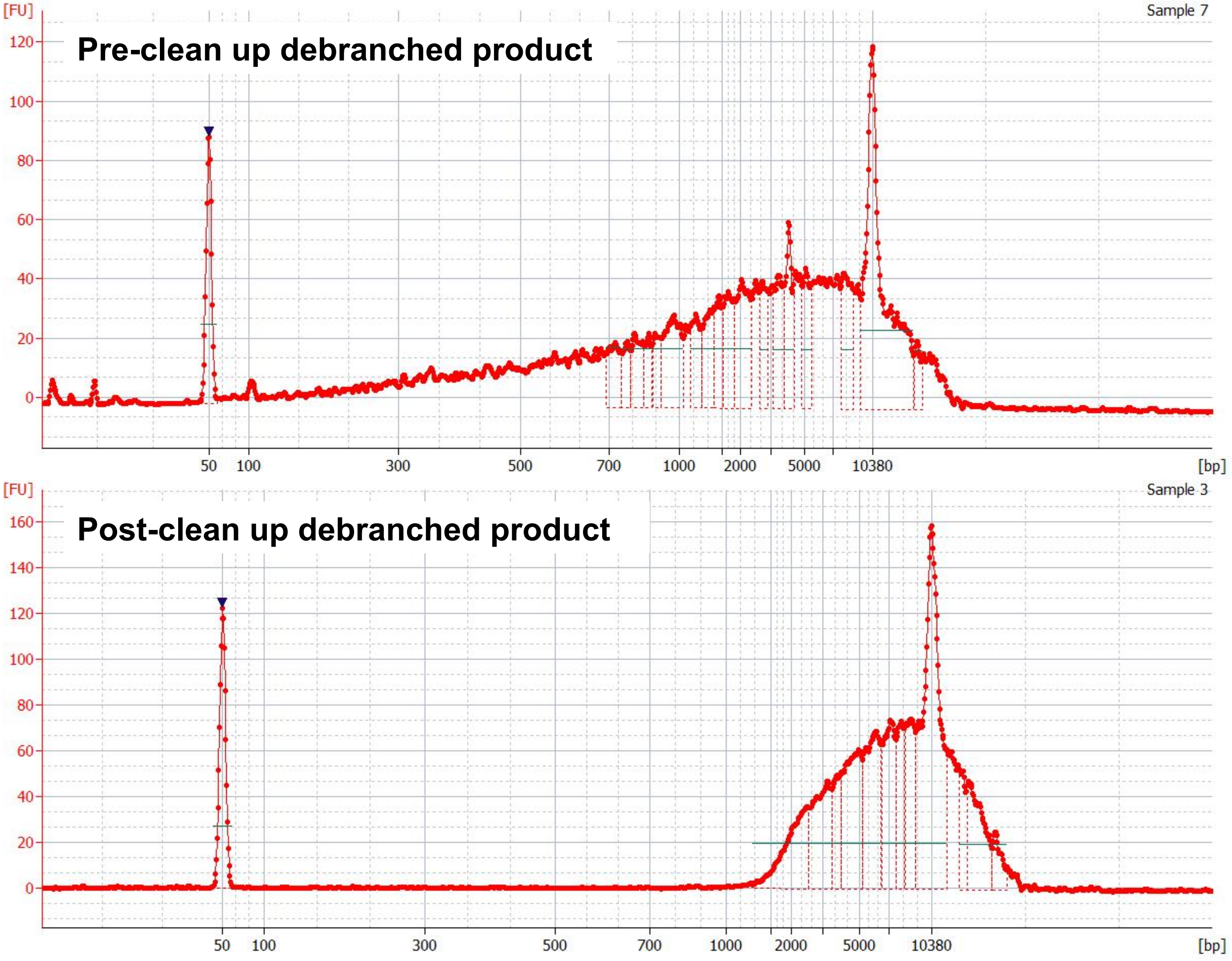

Supplement: Supplemental Files [file giy140_supplemental_files.zip › Supplementary material_R2.pdf]
