# Supplementary material for: NanoAmpli-Seq: a workflow for amplicon sequencing for mixed microbial communities on the nanopore sequencing platform
Source: Gigascience. 2018 Nov 23;7(12):giy140. doi: 10.1093/gigascience/giy140 (PMC6298384; doi:10.1093/gigascience/giy140)
Supplement: response_to_reviewer_comments_original_submission.pdf [file giy140_response_to_reviewer_comments_original_submission.pdf]

Author's Response To Reviewer Comments

Close

Please see the "Personal Cover" for detailed response to reviewer comments and suggestions.

Close
