# Supplementary material for: NanoAmpli-Seq: a workflow for amplicon sequencing for mixed microbial communities on the nanopore sequencing platform
Source: Gigascience. 2018 Nov 23;7(12):giy140. doi: 10.1093/gigascience/giy140 (PMC6298384; doi:10.1093/gigascience/giy140)
Supplement: response_to_reviewer_comments_revision_1.pdf [file giy140_response_to_reviewer_comments_revision_1.pdf]

Author's Response To Reviewer Comments

Close

1. Please add a citation to the amplicon data in GigaDB from the Availability of supporting data section. I would suggest adding an additional line similar to the following:Amplicon data is also included in the GigaScience GigaDB repository[40].

Response: This has been added to the revised manuscript in the "Availability of supporting data
" section as follows: "Intermediate and final fasta files for amplicon sequencing processing workflow are included in the GigaScience GigaDB repository [46]." The citation number is [46] instead of [40] due to change in number of references cited as URL's were removed and replaced by citations.

2. Please register NanoAmpli-Seq in the SciCrunch.org database to receive a RRID (Research Resource Identification Initiative ID) number, and include this in your manuscript. This will facilitate tracking, reproducibility and re-use of your tool. You can then include the RRID number in the Availability and requirements section.

Response: NanoAmpli-Seq has been registered in the SciCrunch.org database and its RRID as been added to the revised manuscript in the "Availability and requirements" section as follows: "RRID: SCR_016710".

3. Add ORCID details for the authors that have them on the title page and change any URLs in the body of the article into citations (OUP production will ask for this so you might as well do it now).

Response: ORCID id's for the authors have been added to the title page of the manuscript and all URL's have been changed to within manuscript citations and appropriate references have been included in the reference list.

Close
