# Supplementary material for: NanoAmpli-Seq: a workflow for amplicon sequencing for mixed microbial communities on the nanopore sequencing platform
Source: Gigascience. 2018 Nov 23;7(12):giy140. doi: 10.1093/gigascience/giy140 (PMC6298384; doi:10.1093/gigascience/giy140)
Supplement: reviewer_2_report_(original_submission) -- Amrita Srivathsan — 8/16/2018 Reviewed [file giy140_reviewer_2_report_(original_submission).pdf]

Reviewer Report

Title: NanoAmpli-Seq: A workflow for amplicon sequencing for mixed microbial communities on the nanopore sequencing platform.

Version: Original Submission **Date:** 8/16/2018

Reviewer name: Amrita Srivathsan

Reviewer Comments to Author:

The authors have improved the manuscript substantially by giving more background information, including more details on the bioinformatics, substantiating some parameters in nanoClust and including the analyses of dataset from Li et al. The improvement on INC-seq protocol is useful, the chopSeq algorithm to reorient the sequences would also be useful and so would the improvement in consensus accuracy but there are limitations with regard to the nanoClust algorithm. Overall I think the authors acknowledge the limitations more clearly in the revised manuscript, which makes it more suitable for publication. Nevertheless, prior to this, some further work/clarifications is/are needed:

Major:

1) It is stated that there is no evidence for multi-species consensus when Li et al. dataset was analysed (L533), but there are OTUs with multiple species (L454). It is unclear how this is possible: Is it because one species is in greater abundance? If the consensus is biased to one species, it should be stated as such as it is dataset dependent. Moreover, I would suggest that the number of OTUs obtained from this analyses is stated somewhere in the paragraph beginning at L450, as one has to look into the details of the figure to get this information.

2) Could it be clarified how the nanoclust consensus was obtained? It is stated it was based on MAFFT-G-INS-I alignment, but I didn't come across a criteria for the calling of consensus (most frequent base, i.e. majority rule?) This will help clarify (1).

3) Figure 7 summarizes how number of partitions affects nanoclust results. The lengths analysed suggest that the partitions are non-overlapping. If so, it is not clear why that would be needed: I think nanoclust would be more useful if it allows for a moving window type of analyses for partitioning the data and the analyses for optimal length would be more informative. Figure 7 suggests that use of full 1300 bp is underestimating the MOTUs while using 650 is overestimating it. Perhaps the optimal solution lies somewhere in between.

4) Lastly, it would be good if the initial coverage of INC-seq reads is included for nanoclust clustering, if this has not been done. This could be checked if one runs vsearch under --sizein parameter and give number of concatamers as size. Nanoclust excludes singletons but the problem is singletons are not exactly singletons, as they are already consensus sequences with some amount of coverage information, depending on how many concatemers were present. If a singleton was based on 42 concatemers vs 3, its accuracy is likely to be different. Part of the problem as to why full length sequences underestimate diversity seems to be because singletons are removed (L362). Running VSEARCH with abundance input might be more helpful in determining which "singletons" are truly of poor quality and ought to be discarded.

Minor:

1) L46 Edit to "average consensus accuracy"

2) Lines 74-77: Avoid repetition of "long-read"

3) L99: The statement incorrect read mapping is prevalent needs a citation. Else it should be rephrased as a possibility.

4) L112: For the "latter" effort?

5) L130-135 is quite long and hard to follow.

6) L136 and throughout text: The "2" in 1D2 should be a superscript.

7) L198: The percent "identity"?

8) L213: Packages within Biopython need not be mentioned.

9) L306-312 is quite hard to follow.

10) L307: Increased from?
11) L359: Do the authors mean to state As the partition size "increased" the number of OTUs decreased and the overall sequence accuracy increased
12) L360: The line "The highest average sequence was observed with a single partition" needs to be corrected

Level of Interest

Please indicate how interesting you found the manuscript: Choose an item.

Quality of Written English

Please indicate the quality of language in the manuscript: Choose an item.

Declaration of Competing Interests

Please complete a declaration of competing interests, considering the following questions:

- Have you in the past five years received reimbursements, fees, funding, or salary from an organisation that may in any way gain or lose financially from the publication of this manuscript, either now or in the future?
- Do you hold any stocks or shares in an organisation that may in any way gain or lose financially from the publication of this manuscript, either now or in the future?
- Do you hold or are you currently applying for any patents relating to the content of the manuscript?
- Have you received reimbursements, fees, funding, or salary from an organization that holds or has applied for patents relating to the content of the manuscript?
- Do you have any other financial competing interests?
- Do you have any non-financial competing interests in relation to this paper?

If you can answer no to all of the above, write 'I declare that I have no competing interests' below. If your reply is yes to any, please give details below.

I declare that I have no competing interests

I agree to the open peer review policy of the journal. I understand that my name will be included on my report to the authors and, if the manuscript is accepted for publication, my named report including any attachments I upload will be posted on the website along with the authors' responses. I agree for my report to be made available under an Open Access Creative Commons CC-BY license (http://creativecommons.org/licenses/by/4.0/). I understand that any comments which I do not wish to be included in my named report can be included as confidential comments to the editors, which will not be published.

I agree to the open peer review policy of the journal

To further support our reviewers, we have joined with Publons, where you can gain additional credit to further highlight your hard work (see: https://publons.com/journal/530/gigascience). On publication of this paper, your review will be automatically added to Publons, you can then choose whether or not to claim your Publons credit. I understand this statement. Yes
