# Supplementary material for: NanoAmpli-Seq: a workflow for amplicon sequencing for mixed microbial communities on the nanopore sequencing platform
Source: Gigascience. 2018 Nov 23;7(12):giy140. doi: 10.1093/gigascience/giy140 (PMC6298384; doi:10.1093/gigascience/giy140)
Supplement: reviewer_3_report_(original_submission) -- Lee Kerkhof — 8/18/2018 Reviewed [file giy140_reviewer_3_report_(original_submission).pdf]

Reviewer Report

Title: **NanoAmpli-Seq: A workflow for amplicon sequencing for mixed microbial communities on the nanopore sequencing platform.**

Version: **Original Submission** **Date:** 8/18/2018

Reviewer name: **Lee Kerkhof**

Reviewer Comments to Author:

The revised manuscript has incorporated many of the suggested editorial comments by the reviewers and is greatly improved. However, there is one fundamental question in their data presentation that would be helpful to address if their methods are to be used for natural samples. Specifically, the NanoAmpli-Seq data analysis pipeline removes self-ligated circles containing different copies of the 16S rRNA genes (<97% similarity; line 176-177). It is unclear how many such "chimeric" circular molecules would be created using a natural sample containing thousands of different microbes or more. There is some sense of sequencing template loss that can be gleaned from Table 1. It would seem these chimeric circles can account for up to 25-58% of the raw reads. But, other factors during analysis (unmappable anchors or short reads) can also account for this loss. It would be good for the authors to provide a sense of the abundance of these "chimeric" circles. For a natural sample, the "chimeric" molecules may account for a much higher percentage of the self-ligated molecules compared with a 10-member system. If the self ligation step for a natural sample yields a significantly large percentages of circular molecules with more than one 16S rRNA gene, then the utility of a NanoAmpli-Seq approach to characterize natural samples (line 139) is brought into question.

Perhaps the authors could also investigate a subset of the "discarded chimeric" reads to see if they may be ultimately brought back into their NanoAmpli-Seq analysis pipeline to alleviate this data loss. This may improve accuracy by providing additional reads for consensus building and make the method more useful for natural samples.

Ultimately, I agree that a method to develop highly accurate sequences de nova from amplicons (such as 16S rRNA genes) without the need for screening against a database would be desirable. However, just because a microorganism's 16S rRNA gene is not well represented in a database does not mean the read is completely ignored (line 101). Virtually all microbiome studies continue to find sequences not well represented in our databases. These novel sequences ultimately become included in databases when enough highly similar reads are discovered and annotated.

Other Specific Comments:

Line 101. It would be more accurate to state that reads not well represented are difficult to unambiguously assign to a single OTU and could lead to clustering errors when widely different 16S rRNA genes hit the same entry in the database at roughly the same similarity.

2). Line 359. Shouldn't that read "As the number of partitions decreased"? As written, the sentence seems to contradict lines 354-356.

3). Line 380-382. The connection from deletions/insertions to the results in Figure 8 are not clear.

4). Line 502. 30x coverage is also consistent with rRNA operon sequencing [ref 20].

Level of Interest

Please indicate how interesting you found the manuscript: Choose an item.

Quality of Written English

Please indicate the quality of language in the manuscript: Choose an item.

Declaration of Competing Interests

Please complete a declaration of competing interests, considering the following questions:

- Have you in the past five years received reimbursements, fees, funding, or salary from an organisation that may in any way gain or lose financially from the publication of this manuscript, either now or in the future?
- Do you hold any stocks or shares in an organisation that may in any way gain or lose financially from the publication of this manuscript, either now or in the future?
- Do you hold or are you currently applying for any patents relating to the content of the manuscript?
- Have you received reimbursements, fees, funding, or salary from an organization that holds or has applied for patents relating to the content of the manuscript?
- Do you have any other financial competing interests?
- Do you have any non-financial competing interests in relation to this paper?

If you can answer no to all of the above, write 'I declare that I have no competing interests' below. If your reply is yes to any, please give details below.

I declare that I have no competing interests

I agree to the open peer review policy of the journal. I understand that my name will be included on my report to the authors and, if the manuscript is accepted for publication, my named report including any attachments I upload will be posted on the website along with the authors' responses. I agree for my report to be made available under an Open Access Creative Commons CC-BY license (http://creativecommons.org/licenses/by/4.0/). I understand that any comments which I do not wish to be included in my named report can be included as confidential comments to the editors, which will not be published.

I agree to the open peer review policy of the journal

To further support our reviewers, we have joined with Publons, where you can gain additional credit to further highlight your hard work (see: https://publons.com/journal/530/gigascience). On publication of this paper, your review will be automatically added to Publons, you can then choose whether or not to claim your Publons credit. I understand this statement. Yes
